# Demographic and Football-Related Characteristics Associated with Objective Cognitive Performance in Former NFL Players with a Normative Sample Comparison: An NFL-LONG Study

**DOI:** 10.1007/s40279-026-02492-7

**Published:** 2026-07-16

**Authors:** Landon B. Lempke, Benjamin L. Brett, Ruben J. Echemendia, Wesley R. Cole, J. D. DeFreese, Danielle L. Hunt, Rebekah C. Mannix, Kevin M. Guskiewicz, Michael A. McCrea, William P. Meehan

**Affiliations:** 1 Department of Physical Medicine and Rehabilitation, Virginia Commonwealth University School of Medicine, 1223 E. Marshall St., Suite 410, Egyptian Building, Richmond, VA 23219 USA; 2 Brain Health Coordinating Center, Richmond Veterans Affairs Medical Center, Richmond, VA USA; 3 Department of Neurosurgery and Neurology, Medical College of Wisconsin, Milwaukee, WI USA; 4 Psychological and Neurobehavioral Associates, Inc, State College, PA USA; 5 University of Missouri-Kansas City, Kansas City, MO USA; 6 University of North Carolina at Chapel Hill, Chapel Hill, NC USA; 7 Division of Sports Medicine, Boston Children’s Hospital, Boston, MA USA; 8 The Micheli Center for Sports Injury Prevention, Waltham, MA USA; 9 Departments of Emergency Medicine and Pediatrics, Harvard Medical School, Boston, MA USA; 10 Division of Emergency Medicine, Boston Children’s Hospital, Boston, MA USA; 11 Department of Kinesiology, Michigan State University, East Lansing, MI USA; 12 Department of Pediatrics, Harvard Medical School, Boston, MA USA

## Abstract

**Background:**

Risk for neurocognitive disorder is a growing concern for contact sport athletes owing to reports associating head impact exposure (HIE) metrics and cognitive function.

**Objective:**

To (1) examine the associations and relative importance between objective cognitive performance, demographics, and HIE metrics of former National Football League (NFL) players and (2) compare cognitive performance between former NFL players and matched samples.

**Methods:**

Cross-sectional analysis of former NFL players (*n* = 295, age: 54.1 ± 14.8 years) using a remote neurocognitive assessment platform (TestMyBrain) collected between March 2022 and October 2022. Players were 1:1 propensity score matched to general population normative male samples on age, race, and highest education level. Cognitive subtests included digit symbol matching (DSM), gradual onset continuous performance test (Grad CPT), trail making test parts A and B (Trails-A -B), paired associates memory for verbal (Verbal PA), paired associates memory for visual (Visual PA), and vocabulary. The HIE metrics examined were age of first exposure to football (AFE), lifetime exposure to football, professional career duration, and lifetime concussion history. Multivariable linear regressions with dominance analysis examined associations and relative importance of demographic data and HIE metrics on cognitive performance; independent *t*-tests examined group differences across cognitive subtests using mean differences (MDs) and 95% confidence intervals (95%CIs).

**Results:**

Age, race, and highest education demonstrated complete dominance over all HIE metrics across the seven cognitive subtests among former NFL players. Professional football career duration demonstrated overall general to complete dominance over the majority of HIE metrics across cognitive subtests, such that greater career duration was associated with lower cognitive performance, while AFE was often dominated by other HIE metrics. Compared to the normative sample, former NFL players had significantly lower DSM (MD: − 3.45, 95% CI − 5.97, − 0.92), Grad CPT (MD: − 14.62, 95% CI − 16.61, − 12.64), Verbal PA (MD: − 5.04, 95% CI − 5.90, − 4.18), Visual PA (MD: − 1.18, 95% CI − 1.90, − 0.46), and vocabulary performance (MD: − 3.84, 95% CI − 5.45, − 2.24), while Trails A and B were not statistically different (*p* ≥ 0.06).

**Conclusions:**

Demographic factors were completely dominant over HIE metrics for cognitive performance. Among only HIE metrics, professional football career duration superseded lifetime concussion history and AFE. Worse cognitive performance was observed among former NFL players compared with a matched normative sample with varying effect sizes.

**Supplementary Information:**

The online version contains supplementary material available at https://doi.org/10.1007/s40279-026-02492-7.

## Key Points

**Table:** 

Demographic factors (age, race, education) were more strongly and consistently associated with cognitive performance than head impact exposure metrics.
Among HIE metrics, professional career duration showed the strongest relative association, although HIE metrics collectively explained comparatively little variance after considering demographic factors.
Former NFL players showed lower performance than a convenience-sampled matched normative group on several cognitive subtests, such as sustained attention and verbal memory, indicating potential domain-specific differences warranting further evaluation.

## Introduction

American tackle football is one of the most common sports among male youth athletes in the USA [1]. However, the head impact exposure (HIE) experienced by athletes playing football and potential long-term negative health effects have led to growing concerns regarding safety and long-term consequences [2–5]. Of particular concern has been the long term cognitive functioning of National Football League (NFL) players, as they receive a relatively large amount of HIE. Research to date has observed mixed evidence regarding whether former NFL players are at greater risk for cognitive impairment, dementia, and other neurologic conditions compared with the general population [6–10]. Beyond neurologic disease, studies have also used cognitive tests measuring different functional domains via self-report [11–14] or objective measures [15–18] also with heterogeneous collective findings. Notably, there is often a disconnect between subjective self-perception of cognitive functioning and objective cognitive performance [18, 19] that complicates efforts to synthesize findings and determine the extent of cognitive effects in former NFL players. Because subjective concerns and objective performance related to cognition may diverge, objective testing provides a complementary approach for evaluating cognitive outcomes in former NFL players.

Levels of cognitive performance are broadly associated with a range of demographic and health-related factors such as age, race, ethnicity, sleep quality, cardiovascular health, crystalized intelligence, and concussion history [20, 21]. However, the extent to which these factors and others collectively contribute to cognitive performance in former NFL players remains unclear [11, 15–18, 21, 22]. Mixed evidence across studies can be partially attributed to heterogeneity in participant recruitment and disparate inclusion criteria [23, 24].

Prior research among football players has also commonly examined the associations of individual HIE metrics such as concussion history, total years of football participation, and age at first exposure (AFE) in isolation [13–16, 22, 25, 26]. These HIE metrics are typically multicollinear (e.g., more concussions are associated with more years of play; earlier AFE is associated with longer career duration) [14, 25], often introducing analytic challenges to isolating the independent effect of each HIE metric using traditional general linear models. More advanced statistical approaches, such as dominance analysis [27, 28], can address correlated variables and partition explained variance across each HIE metric to identify which has the strongest relative contribution to cognitive performance. Identifying the specific HIE metric(s) driving associations with cognitive performance is important for understanding risk factors and potential intervention targets, but to date HIE metrics have not thoroughly been examined in this manner.

In addition, some prior studies indicate lower cognitive performance among former contact sport athletes compared with controls [16], while others report minimal [17] or no significant differences [18, 29]. One recent study [16] examined the associations between HIE metrics and cognitive performance using a remote test battery with 353 former NFL players. Cognitive performance was compared with an age-matched general population sample and observed that former NFL players had statistically worse cognitive performance on six different subtests (Trails A and B, digit symbol matching, multiple object tracking, verbal and visual paired associates), though effect sizes were small (*r*_p_ range: − 0.19, − 0.07). However, demographic factors beyond age were left unexamined and may have affected cognitive performance. Therefore, there remains uncertainty regarding the nature of cognitive performance and noteworthy demographic and HIE metrics that relate to cognition in former NFL players, thus warranting further study.

To address these limitations, we sought to: (1) examine associations and the relative importance between objective cognitive performance, demographic characteristics, and HIE metrics of former NFL players, and (2) compare cognitive performance between former NFL players and an age-, race-, and education-matched sample from the general population.

## Methods

We conducted a cross-sectional analysis of former NFL players enrolled in a larger, multiphasic longitudinal project called, Neurologic Function across the Lifespan: A Prospective, LONGitudinal, and Translational Study for Former National Football League Players (NFL-LONG), detailed elsewhere [30–33]. In brief, former NFL players were recruited via email or mailed letters sent through listservs from the NFL Alumni Association (an independent support and care organization from the NFL) or to individual teams or players through existing connections to complete a general health questionnaire with honorarium provided for completing. For this analysis, data collected between January 2019 and February 2020 were analyzed.

### Participants

Players were included if they played in at least one NFL season. A total of 2,030 hard copy or electronic general health questionnaires were collected, with 236 excluded for being duplicate records from players and an additional 10 players excluded for not providing identifying information, leaving a total of 1784 former NFL players [33]. Completion of the questionnaire after the introductory paragraph implied consent. All study procedures were reviewed and approved by the IRBs at the University of North Carolina-Chapel Hill, Medical College of Wisconsin, and Boston Children’s Hospital.

Specific to this report, the 1784 former NFL players were invited between March 2022 and October 2022 to participate in a remote, objective neurocognitive evaluation sub-study using the TestMyBrain platform [16, 34]. A total of 342 former NFL players initially completed the remote neurocognitive evaluation. We stepwise excluded former players who did not complete TestMyBrain in a single session (*n* = 35), completed it on a mobile device (*n* = 4; owing to unknown performance differences relative to computer-based), or reported Alzheimer's disease or related dementias (*n* = 8) leaving *n* = 295 in the final sample.

For the normative matched sample, we used participant-level normative data provided by TestMyBrain to perform 1:1 nearest neighbor propensity score matching[35] on age, race, and education to control for established confounders where common variables in the former player and normative sample were available for each cognitive test separately (i.e., seven separate matched normative samples). Importantly, race was included as a self-reported variable and should not be interpreted as reflecting biological differences in cognitive performance, but rather likely reflecting a proxy of the cumulative structural, social, educational, medical, and interpersonal factors not directly measured in this study [36]. Propensity score matching was used as a design-stage approach to improve comparability on these observed demographic characteristics for each cognitive test separately (i.e., seven separate matched normative samples). Propensity score match balancing was evaluated and confirmed for groups across age, race, and education by evaluating the absolute standardized mean differences (≤ 0.10 threshold) between the matched and unmatched samples. Prior to propensity score matching, we excluded individuals from the normative matched sample group if they did not complete TestMyBrain in a single session, on a mobile device, were outside the USA, or English was not their primary language. Owing to how normative general population data were collected and stored within the neurocognitive platform, it was not possible to link individual participant scores across subtests [16, 34]. By contrast, scores from the former NFL player sample were explicitly linked across subtests.

### General Health Questionnaire

The self-administered General Health Questionnaire was adapted and modified from a previously established questionnaire used among former NFL Players [37, 38] designed to comprehensively assess demographics (e.g., age, race, education), detailed football playing and concussion history (i.e., HIE metrics), medical history and diagnoses, musculoskeletal injury history, current health status, current nutrition behaviors, substance use, current health-related habits, and self-reported cognitive, behavioral, and physical function [33]. Overarching General Health Questionnaire modifications included for the NFL-LONG study were related to evaluating a broader range of health-related domains surrounding neurobehavioral, psychosocial, lifestyle, and health behavior construct, and refinement of football participation, injury history, and medical history items. Content revisions were guided by a multidisciplinary team with expertise in neuropsychology, athletic training, epidemiology, general medicine, exercise science, and sports medicine. Five former NFL players also piloted, reviewed, and provided feedback to ensure clear question and response formatting.

In this analysis, demographic characteristics, health history, HIE metrics, were evaluated. Specific demographic characteristics included age at the time of remote cognitive evaluation, self-reported race (including the option to report more than one race), and highest education level. Participants reported whether a physician or other healthcare provider told them they have or had any of the following separate diagnoses: anxiety, depression, ADD/ADHD, learning disorder, or mild cognitive impairment.

The HIE metrics of (1) lifetime concussion history, (2) AFE, (3) lifetime football exposure, and (4) professional career duration were evaluated owing to their common examination in the literature [13–16, 22, 25, 26]. As with prior studies, former players were given the following definition of concussion: “A concussion typically occurs from a blow to the head and is followed by a variety of symptoms that may include any of the following: headache, dizziness, loss of balance, blurred vision, ‘seeing stars’, feeling in a fog, or slowed down, memory problems, poor concentration, nausea, or throwing-up. Getting ‘knocked out’ or being unconscious does NOT always occur with a concussion.” They were then asked to self-report all potential concussions they experienced while playing football, even if they were not medically diagnosed [33]. Lifetime concussion history was evaluated as a discrete variable. AFE was derived from the survey item asking participants “At what age (in years) did you start playing organized football?” and was evaluated as a discrete variable in line with prior reports [14] and recommended statistical practices to minimize spurious findings [39, 40]. Lifetime football exposure was derived through participant responses to items asking start and end years of football participation during youth, high-school, college, and professional timeframes, and the difference between the latest and earliest years reported. Professional football career duration was derived from the same participant response, but only from the start and end years reported for their professional football career.

### Remote Neurocognitive Testing

The remote, computerized TestMyBrain neurocognitive platform is a freely available battery with established psychometrics [16, 34] using foundational neuropsychological tests[34] to reliably evaluate verbal and visual memory, working memory, processing speed, attention, and vocabulary. Completions were evaluated for time and device completion validity (i.e., completed in one sitting, on same device, not on a mobile device, in established normative time limit) to ensure data quality and testing protocol compliance.

Cognitive subtests consisted of digit symbol matching (DSM), gradual onset continuous performance test (Grad CPT), trail making test parts A (Trails A) and B (Trails B), paired associates memory for verbal (Verbal PA) and paired associates memory for visual (Visual PA), and vocabulary. The DSM evaluated participants’ ability to correctly match pairs of shapes and numbers, with higher scores reflecting accurate responses and indicate faster processing speed. The Grad CPT required participants to concentrate on a continuous stream of city and mountain scenes to evaluate how accurately they were able to make responses to city scenes while withholding responses to mountain scenes, with higher scores reflecting better sustained attention and cognitive control. Trails A and Trails B involved participants connecting target dots as quickly as possible, with participants sequentially connecting dots using number labels (e.g., 1–2-3–4) for Trails A, and connecting dots by alternating between number and letter labels (e.g., 1-A-2-B) for Trails B. Each Trails A and B score reflected how many correct connections between dots they made per minute with higher scores reflecting psychomotor speed (Trails A) and executive function (Trails B) [16]. Verbal PA required participants to learn and remember word pairs based on the number of word pairs they correctly identified out of 25, with higher scores indicating better verbal memory. Visual PA evaluated participants’ ability to learn and remember image pairs on the basis of the number of image pairs they correctly identified out of 24, with higher scores indicating better visual memory. Vocabulary was adapted from the General Social survey Wordsum test [41] where participants decided which of five words was closest in meaning to target word. Each participant’s score is the number of words they correctly defined. Higher scores indicate better verbal knowledge.

### Statistical Analysis

Descriptive statistics (means and standard deviations, medians and 25th–75th percentiles, frequencies and proportions) were conducted for former NFL players by age, race, education, AFE, lifetime football exposure, professional career duration, lifetime concussion history, and self-reported diagnoses and compared relative to the full sample completing the General Health Questionnaire to understand potential sampling bias. Descriptives for the normative matched sample was provided by age, race, and highest education. Analyses used raw cognitive scores rather than standardized values to preserve the scale interpretability of each test’s metric (e.g., accuracy or number correct) and avoid introducing sample-specific transformation artifacts.

We used seven separate dominance analyses to determine the relative importance of demographic and HIE metrics in explaining variability among each of the seven cognitive subtests, using the DominanceAnalysis package in R [42]. Specifically, the outcome variable for each model was each cognitive subtest raw score, with explanatory variables including HIEs (AFE, lifetime football exposure, professional career duration, lifetime concussion history) and demographics (age, highest education level, race). Only these static demographic factors were included rather than other established modifiable lifestyle or health behavior factors associated with cognitive performance (e.g., diet, exercise, sleep, smoking/drinking habits) [33, 43]. These lifestyle variables were assessed during the initial General Health Questionnaire, which was completed 2–3 years before the remote cognitive evaluation. Because these behaviors are time-varying, including them would require the unverifiable assumption that they remained stable between questionnaire completion and cognitive testing. We therefore did not include these variables in the primary dominance models.

All dominance analyses were specified with base linear regression models. Dominance analyses then proceeded by estimating all possible additive subset regressions and determining the incremental contribution of each predictor to variance explained (coefficient of determination; *R*^2^) as the criterion metric. Dominance analyses are well-suited for parsing out relative contribution to *R*^2^ for predictors with theorized or established multicollinearity [27, 28], such as among the numerous HIE metrics present in the literature and evaluated here [14, 25]. Each predictor’s average contribution across all possible model subsets was calculated (i.e., general dominance), as well as its contribution in models of a given size (i.e., conditional dominance) and when directly compared with another predictor across all possible models (i.e., complete dominance). In brief, predictors demonstrating complete dominance indicate contributing greater *R*^2^ than another predictor in every possible model and is the most stringent criterion. Conditional dominance demonstrates the average increase in *R*^2^ associated with a predictor when it is included at a specific position in the additive sequence of predictors. Lastly, general dominance reflects the relative ranking of predictors averaged *R*^2^ contribution across averaged model subsets. All dominance analyses were bootstrapped with 1000 replications in accordance with Azen and Budescu procedures [27] to evaluate consistency and reliability of dominance criteria observed across all models.

For the former NFL player and normative sample cohort comparison, propensity score matching was used as the primary design-stage approach to account for age, race, and highest education. Because matching was used for comparable former NFL players and normative sample groups for each cognitive subtest, the post-matching analysis focused on direct group comparisons of raw cognitive scores rather than multivariable models estimating a conditional regression coefficient. Independent *t*-tests were therefore used to examine post-matching differences between former NFL players and the age-, race-, and education-matched normative sample groups across all cognitive subtests. Mean differences (MDs) with 95% confidence intervals (95% CIs) were calculated to provide unstandardized effect estimates on the natural cognitive subtest scale. Cohen’s d effect sizes were also calculated to provide supplemental standardized effect estimates. All model assumptions were visually assessed via graphical plots and confirmed prior to reporting. Statistical analyses were performed using the R Foundation for Statistical Programming (version 4.2.4; Vienna, Austria) with *α* = 0.05 a priori.

## Results

The final sample was middle-aged, predominately white, and had a bachelor’s degree as their highest education level attained (Table 1). Former NFL players had a median of four lifetime self-reported concussions and average lifetime football exposure of 11.52 years, with an average of 5.11 years of professional football (Table 1). Compared with the full NFL-LONG sample of 1784 former players, the sample in this study had a significantly higher proportion of white players, shorter professional football careers (mean difference = 0.72 years), more lifetime concussions (mean difference = 0.54), and lower (7%) self-reported prevalence of mild cognitive impairment (Table 1). The remote cognitive sample did not significantly differ from the full NFL-LONG sample by age, highest education level, AFE, lifetime football exposure, or by proportion of players with self-reported psychological health or learning disorders. The former NFL player sample was appropriately 1:1 nearest neighbor propensity matched to the normative samples as demonstrated in Table 2 with comparable age, race, and highest education characteristics.

**Table 1:** Demographic comparison of former NFL players by TestMyBrain completion

	TestMyBrain sample, *N* = 295	Full sample, *N* = 1784	*p*-value^a^
Age at evaluation (years)			0.057
Mean (SD)	54.10 (14.77)	52.34 (16.32)	
Median (IQR)	54.00 (41.00–67.50)	53.00 (37.00–66.00)	
Race			0.002
Asian/Pacific Islander	1 (0.34%)	13 (0.73%)	
Black	73 (24.75%)	631 (35.55%)	
Multiracial	7 (2.37%)	47 (2.65%)	
Native American	1 (0.34%)	4 (0.23%)	
Other race	2 (0.68%)	28 (1.58%)	
White	211 (71.53%)	1052 (59.27%)	
Skipped/unknown	–	9	
Highest education			0.12
Associate	9 (3.05%)	49 (2.75%)	
Bachelor	173 (58.64%)	1089 (61.11%)	
Post-graduate	88 (29.83%)	425 (23.85%)	
Some college, no degree	25 (8.47%)	214 (12.01%)	
High school/GED	–	5 (0.28%)	
Skipped/unknown	–	2	
Age of first exposure to football (years)			0.6
Mean (SD)	11.18 (2.85)	11.26 (2.90)	
Median (IQR)	12.00 (9.00–13.00)	12.00 (9.00–14.00)	
Skipped/unknown	8	53	
Lifetime football exposure			0.3
Mean (SD)	11.52 (3.47)	11.42 (3.85)	
Median (IQR)	11.00 (9.00–14.00)	11.00 (9.00–14.00)	
Professional career duration (years)			0.001
Mean (SD)	5.11 (3.21)	5.83 (3.58)	
Median (IQR)	4.00 (3.00–7.00)	5.00 (3.00–8.00)	
Skipped/unknown	3	33	
Lifetime concussion history			0.038
Mean (SD)	5.53 (4.91)	4.99 (4.69)	
Median (IQR)	4.00 (2.00–8.00)	4.00 (1.00–7.00)	
MCI reported	35 (11.86%)	337 (18.89%)	0.004
Depression reported	57 (19.32%)	357 (20.01%)	0.8
Anxiety reported	51 (17.29%)	333 (18.67%)	0.6
Learning disorder reported	12 (4.07%)	66 (3.70%)	0.8
ADD/ADHD reported	26 (8.81%)	145 (8.13%)	0.7

^a^Wilcoxon rank sum test; Fisher’s exact test for count data with simulated *p*-value (based on 2000 replicates); Pearson's Chi-squared test

**Table 2:** Former NFL player and matched normative sample demographics

	Former NFL players *N* = 295	Normative sample *N* = 295
Age at evaluation (years)		
Mean (SD)	54.10 (14.77)	55.33 (16.17)
Median (IQR)	54.00 (41.00–67.50)	58.00 (41.00–69.00)
Race		
Asian/Pacific Islander	1 (0.34%)	–
Black	73 (24.75%)	52 (17.63%)
Multiracial	7 (2.37%)	5 (1.69%)
Native American	1 (0.34%)	1 (0.34%)
Other race	2 (0.68%)	–
White	211 (71.53%)	237 (80.34%)
Highest education		
Associate	9 (3.05%)	3 (1.02%)
Bachelor	173 (58.64%)	178 (60.34%)
Post-graduate	88 (29.83%)	90 (30.51%)
Some college, no degree	25 (8.47%)	24 (8.14%)
DSM (no. correct)		
Mean (SD)	56.01 (17.03)	59.46 (14.03)
Median (25th–75th)	58.00 (45.00–69.50)	60.00 (51.00–69.00)
Grad CPT (accuracy, %)		
Mean (SD)	69.00 (15.65)	83.63 (7.54)
Median (25th–75th)	71.87 (59.37–81.25)	84.78 (79.22–89.16)
Trails A (response rate)		
Mean (SD)	42.53 (21.72)	45.26 (21.55)
Median (25th–75th)	40.01 (24.43–57.08)	41.85 (27.86–60.48)
Trails B (response rate)		
Mean (SD)	29.76 (13.97)	31.98 (14.74)
Median (25th–75th)	28.08 (19.08–37.47)	30.45 (22.06–39.23)
Verbal PA (no. correct)		
Mean (SD)	13.41 (5.59)	18.44 (5.02)
Median (25th–75th)	13.00 (9.00–17.50)	19.00 (15.00–23.00)
Visual PA (no. correct)		
Mean (SD)	13.42 (4.42)	14.59 (4.47)
Median (25th–75th)	13.00 (11.00–16.00)	14.00 (11.00–18.00)
Vocabulary (accuracy, %)		
Mean (SD)	85.94 (10.87)	89.79 (8.91)
Median (25th–75th)	90.00 (83.33–93.33)	93.33 (86.67–96.67)

### Dominating Factors Associated with Cognitive Performance in Former NFL Players

**Fig. 1:**
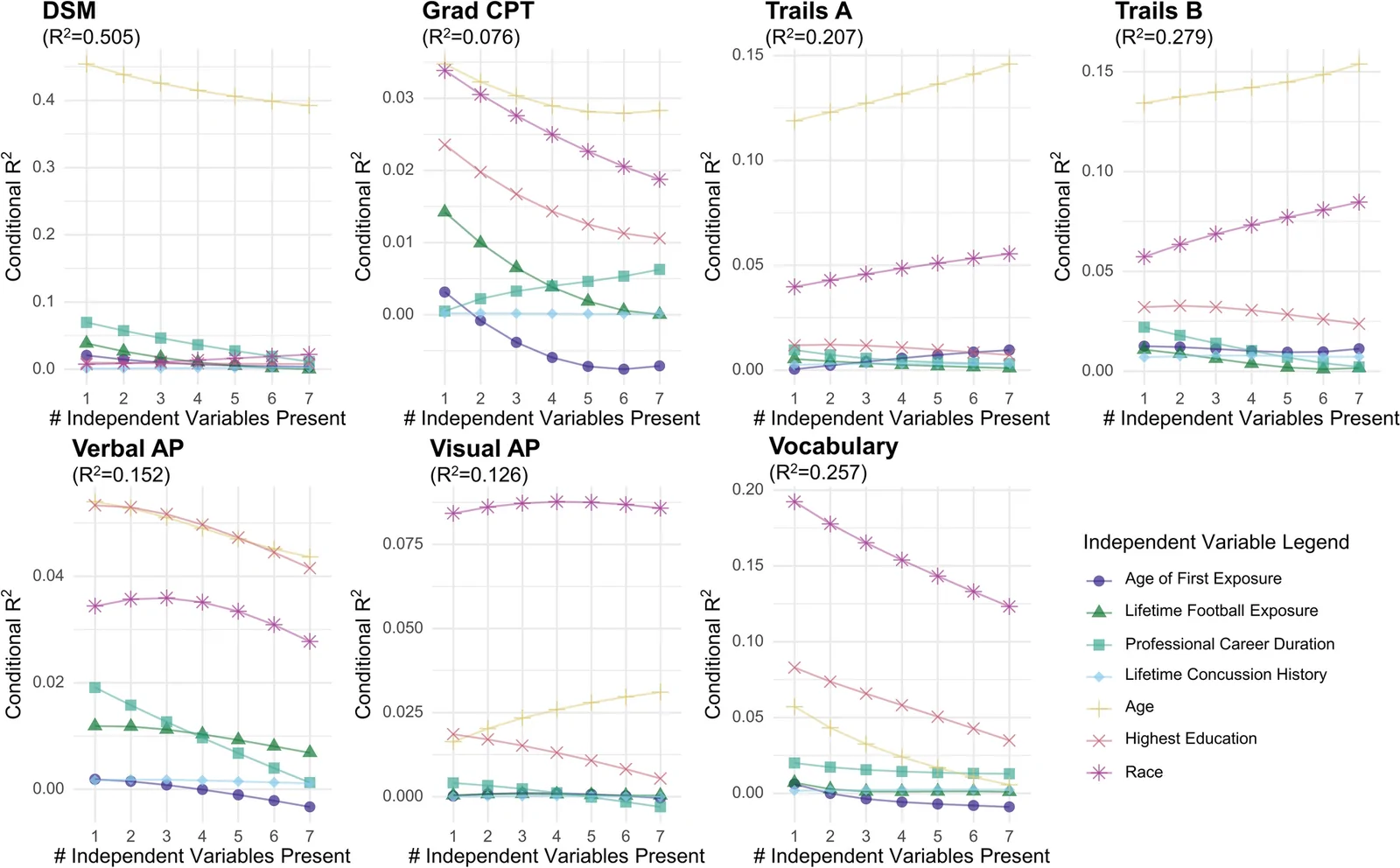
Dominating factors associated with cognitive performance in former NFL players (*n* = 295). Dominance analysis results with 1000 bootstrap replications for the seven TestMyBrain cognitive subtests. Each panel depicts conditional dominance values, expressed as coefficient of determination values (*R*^2^; *y*-axis), from all possible subset linear regression models. The *x*-axis represents the number of independent variables included in the subset models. For each independent variable, the plotted value represents its average incremental contribution to explained variance (*R*^2^) when added to models of a given size. Thus, larger *R*^2^ values indicate greater relative importance for explaining variability in the corresponding cognitive subtest across model combinations of that size. These values indicate the relative contribution of each demographic or head impact exposure metric to model-explained variance. *DSM* digit symbol matching, *Grad CPT* Gradual Onset Continuous Performance Test, *Trails A* Trail Making Test part A, *Trails B* Trail Making Test part B, *Verbal PA* Verbal Paired Associates Memory, *Visual PA* Visual Paired Associates Memory

Dominance analysis with bootstrapping conducted on all seven cognitive subtests revealed that demographic factors of age, race, and highest education were the most consistently dominant predictors of subtest performance (Fig. 1; Supplementary Table 1). Specifically, age demonstrated complete dominance over all other demographics and HIE metrics for DSM, Grad CPT, Trails A, and Trails B. Race demonstrated complete dominance over all other demographics and HIE metrics for Visual PA and Vocabulary. No demographic or HIE metric demonstrated complete dominance over all others for Verbal PA. Bootstrapping evaluation overall demonstrated low standard error estimates with strong replication, and stable, consistent dominance for age, highest education, and race across cognitive subtest models (Supplementary Table 2).

Among the HIE-specific predictors, the overall *R*^2^ was low relative to demographics and the cumulative model (Fig. 1). There were mixed findings regarding the strongest dominating HIE metric across all cognitive subtests, with less stability and replication via bootstrap models (Supplementary Table 2). Across all subtests, professional football career duration was the strongest relative HIE metric with complete dominance over lifetime concussion history for DSM and Grad CPT, and over AFE for all seven cognitive subtests (Fig. 1). Lifetime concussion history demonstrated complete dominance over AFE for all cognitive subtests except DSM and Grad CPT. Across subtests, AFE was commonly dominated by other HIE metrics with no instances of completely dominating other HIEs across all cognitive domains. Lifetime football exposure did not demonstrate complete dominance over, nor was it completely dominated by, any HIE metric across models (Fig. 1, Supplementary Table 1).

### Cognitive Performance Between Former NFL Players and Normative Matched Sample

**Fig. 2:**
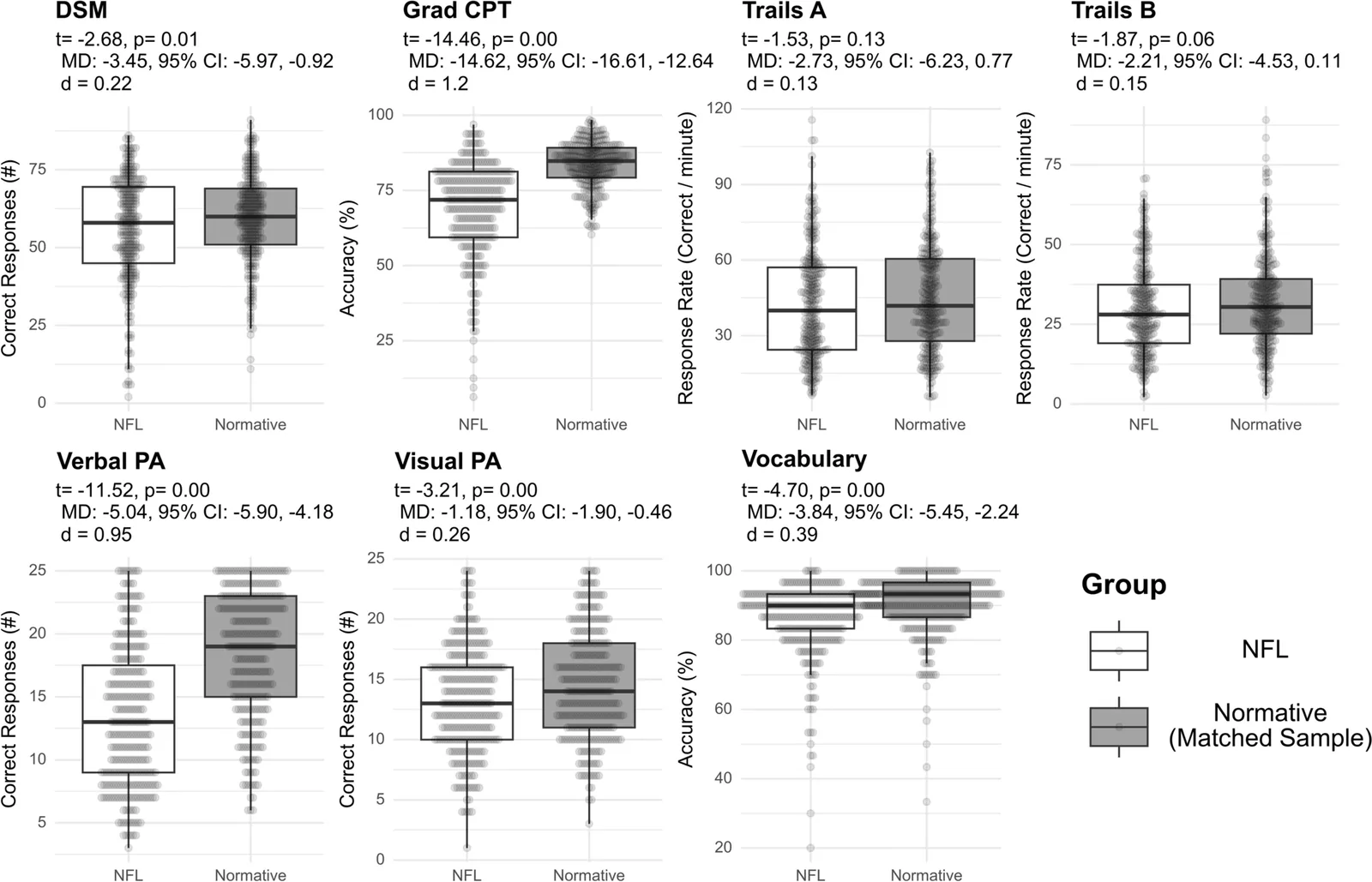
Cognitive performance between former NFL Players and matched normative sample. Tukey boxplots with overlaid bee swarm plot of individual datapoints (10% transparency) depicting data distribution and summary statistics for each group (former NFL players = white, normative matched sample = gray). Each panel is separated for the seven TestMyBrain cognitive subtests, with accompanying *t*-statistic, mean differences (MD; normative matched sample = referent) with accompanying 95% confidence intervals (95% CI), and Cohen’s d (*d*) effect sizes from the independent *t*-tests. *DSM* digit symbol matching, *Grad CPT* Gradual Onset Continuous Performance Test, *Trails A* Trail Making Test part A, *Trails B* Trail Making Test part B, *Verbal PA* Verbal Paired Associates Memory, *Visual PA* Visual Paired Associates Memory

Compared with the normative-matched samples, former NFL players had significantly lower cognitive performance across five of the seven subtests (*p* ≤ 0.05; Table 2 and Fig. 2). Specifically, former NFL players had significantly lower (worse) scores with small to large effect sizes on DSM (MD: − 3.45, 95% CI − 5.97, − 0.92, *d* = 0.22), Grad CPT (MD: − 14.62, 95% CI − 16.61, − 12.64, *d* = 1.2), Verbal PA (MD: − 5.04, 95% CI − 5.90, − 4.18, *d* = 0.95), Visual PA (MD: − 1.18, 95% CI − 1.90, − 0.46, *d* = 0.26), and Vocabulary subtests (MD: − 3.84, 95% CI: − 5.45, − 2.24, *d* = 0.39). Both Trails A and Trails B were not significantly different between former NFL players and the normative matched sample (*p* ≥ 0.06; Fig. 2).

## Discussion

Our findings demonstrate that age, race, and highest education level were the most important factors associated with cognitive performance across a range of neurocognitive subtests. Among the cohort of former NFL players, these demographic indices completely dominated all HIE metrics evaluated. Among the HIE metrics, professional football career duration showed the greatest relative impact, frequently dominating other HIE measures, although it showed overall weak associations with cognitive performance in former NFL players. Additionally, we observed statistically worse cognitive performance for former NFL players relative to a matched sample across all subtests except Trails-A and -B, with Grad CPT and Verbal PA demonstrating large magnitude effect sizes of potential clinical relevance. Taken together, our findings indicate cohort differences in cognitive performance may exist, but within former NFL players, HIE metrics accounted for comparatively little variance in objective cognitive performance after considering demographic factors.

Though often proxy measures representing multifactorial constructs, the contributions of age, race/ethnicity, and education attainment on cognitive performance are well-documented [44–46] and consistent with our present findings (Fig. 1). In nearly all dominance analysis models across the cognitive subtests, these three demographic factors displayed general to complete dominance over the HIE metrics with age having the greatest relative importance for processing speed, executive function, attention, and verbal memory, while race was of greatest relative importance for visual memory and vocabulary (Fig. 1). Specifically, these demographic factors consistently dominated AFE, lifetime concussion history, lifetime football exposure, and professional football career duration and underscore the substantially greater weight these demographic characteristics have for cognitive performance over HIE metrics. Future longitudinal work with more comprehensive multidimensional neuropsychological evaluations is warranted to better understand the nature and strength of these associations.

When comparing HIE metric dominance among themselves, the cognitive subtest models were inconsistent in the relative importance of individual HIE metrics (Fig. 1, Supplementary Table 1) and through bootstrapping features (Supplementary Table 2). Although a single HIE metric did not completely dominate the other HIEs, professional football career duration demonstrated the greatest relative dominance over other HIE metrics and indicates the cumulative exposures garnered through professional football, though nonspecific to repetitive head impacts or concussion, is of greatest cognitive relevance. Additionally, AFE was often conditionally or completely dominated by other HIE metrics across cognitive subtests and indicates overall weak relevance and associations with cognitive performance. Early research among former NFL players [22, 47] indicated AFE was associated with cognitive impairment among a cohort of cognitive-, behavioral-, and/or mood-symptomatic former NFL players when dichotomized into age < 12 or ≥ 12 years AFE, with those in the < 12 years group showing poorer performance than the ≥ 12 years group. However, our present findings and the extant AFE literature with broader samples have found either no association or associations with small effect sizes [12, 48–55]. Our findings help underscore and expand the existing research by indicating some HIE metrics, such as AFE, are less informative than other HIE metrics which can provide greater granularity among these inter-related variables. By disentangling these co-occurring HIEs and their relationship to cognitive performance factors, future injury prevention strategies could be developed to target the most deleterious HIE metrics with research validating our findings among other athletic cohorts.

Our former NFL player cohort was similar in age to Strong et al.’s [16] cohort (54.1 ± 14.8 versus 54.3 ± 13.4 years) and demonstrated broadly overlapping TestMyBrain performance across each domain. From a descriptive visual comparison of cohort means, our cohort showed lower Trails A/B response rates, similar Visual PA and Vocabulary performance, and higher DSM and Verbal PA scores. However, substantial overlap in standard deviations was present and indicates likely overall similar cohort performance. Our findings also revealed that former NFL players performed statistically lower across most cognitive subtests relative to normative-matched samples (Fig. 2). Group differences were evident on measures of attention, processing speed, and verbal and visual memory, but not on psychomotor speed or executive function subtests. Specifically, the largest differences were observed on the Grad CPT (MD = − 14.62%; *d* = 1.20) where former NFL players had a 14.62% lower mean accuracy versus the matched sample and for Verbal PA (MD = − 5.04; *d* = 0.95) where former NFL players had 5.04 lower mean correct responses. These findings indicate large unstandardized and standardized negative performance effects for sustained attention and verbal memory among former NFL players. These findings partially align with prior work reporting omnibus differences in cognitive performance among former NFL players, but the specific subtests showing impairment varied. A recent study[16] using the TestMyBrain platform similarly identified worse Visual PA, Verbal PA, and DSM performance among former NFL players relative to age-matched normative samples, yet, contrary to the present study, also reported Trails A and B differences and no differences on Grad CPT or Vocabulary. The Vocabulary subtest reflects crystallized intelligence and may indicate premorbid differences between former NFL players and the normative samples rather than reflecting football career-related exposures. Moreover, the normative reference samples lacked detailed demographic and health history data, which may introduce additional residual confounding and limit interpretability [20, 21]. Taken together, our findings contribute to growing evidence indicating that former NFL players may exhibit lower cognitive performance on some subtests when matched to normative samples based on simple demographics, but football-related HIE metrics demonstrate weak associations to cognitive performance that are dominated by demographic factors. Longitudinal studies designed to identify contributing factors with greater specificity to cognitive changes over time are needed to better understand these complex and dynamic relationships.

Our study had inherent limitations. The demographic health history and HIE metrics were self-reported by the participants and, though these potential limitations are consistent with the majority of existing former NFL player health literature, may be inaccurate given historically poor concussion diagnostic capabilities compared with today. The self-report concern may be most relevant for retrospective HIE metrics, particularly lifetime concussion history. Future studies linking objective exposure records, medical records, and longitudinal cognitive testing are needed to reduce potential recall-related bias.

An additional limitation is the NFL player sample completing the remote cognitive evaluation statistically differed from the larger cohort by race, professional football career duration, concussion history, and self-reported mild cognitive impairment, which may reflect potential sampling biases, though these significant effects were generally small (Table 1). We excluded participants who completed remote cognitive evaluations on mobile devices or did not complete the battery in a single session. However, the evaluation was completed in the participant’s own uncontrolled environment in a remote setting, which may have introduced additional performance variability [56]. Standardized scores were unavailable due to lack of peer-reviewed published normative data with only raw scores published on a sample approximately half the age (mean of 28.8 years old) relative to our former NFL player sample [34]. We used Cohen’s d to represent standardized magnitude differences relative to the study sample distribution, but this does not compare with population norms, which should also be considered when interpreting for clinical magnitude and impairments [57]. Lastly, individual linkage of the TestMyBrain normative participants across subtests was not possible owing to data collection and recording procedures [16], which required seven samples that may or may not have included the same participants. This ultimately may have affected our findings owing to unknown motivations for completing TestMyBrain and unknown health histories. Propensity score matching can improve comparability of observed variables included in the matching procedure but does not address unmeasured differences between cohorts. Therefore, residual bias related to health status, selection into cognitive testing, or motivation for completing TestMyBrain may have impact the former NFL player versus normative sample comparisons.

## Conclusions

Our findings indicate that among former NFL players, age, education, and race have a more robust and consistent association with cognitive performance than AFE, lifetime football exposure, professional career duration, and lifetime concussion history. Although professional career duration was the HIE metric with the strongest relative association with cognitive performance, its effects were small and inconsistent across domains, and no HIE metrics were a dominant factor relating to cognitive performance. Former NFL players displayed statistically lower scores on measures of processing speed and visual memory with large effects for lower sustained attention and verbal memory compared with matched-normative samples. These collective findings emphasize the need for clinicians and researchers to consider multidimensional factors beyond HIE metrics when assessing cognition among former NFL players and underscores the need for future longitudinal studies to clarify the pathways related to long-term cognitive functioning.

## Supplementary Information

Below is the link to the electronic supplementary material.

Supplementary file1 (PDF 476 KB)

## Declarations

### Availability of Data and Material

The dataset is not available for public sharing or upon individual’s reasonable requests owing to privacy and sharing restrictions from the IRB.
