# Supplementary material for: Demographic and Football-Related Characteristics Associated with Objective Cognitive Performance in Former NFL Players with a Normative Sample Comparison: An NFL-LONG Study
Source: Sports Med. 2026 Jul 16;56(12):3155–67. doi: 10.1007/s40279-026-02492-7 (PMC13759642; doi:10.1007/s40279-026-02492-7)
Supplement: Supplementary file 1 [file 40279_2026_2492_MOESM1_ESM.pdf]

Supplementary File

Title: Demographic and Football-Related Characteristics Associated with Objective Cognitive Performance in Former NFL Players with A Normative Sample Comparison: An NFL-LONG Study.

Journal: Sports Medicine

Authors: Landon B. Lempke, PhD, ATC[1,2]; Benjamin L. Brett, PhD[3]; Ruben J. Echemendia, PhD[4,5]; Wesley R. Cole, PhD[6]; J. D. DeFreese, PhD[6]; Danielle L. Hunt, MS, ATC[7,8]; Rebekah C. Mannix, MD, MPH[9,10]; Kevin M. Guskiewicz, PhD, ATC[11]; Michael A. McCrea, PhD, ABPP[3]; William P. Meehan III, MD[7,8,12]

[1] Department of Physical Medicine and Rehabilitation, Virginia Commonwealth University School of Medicine, Richmond, VA, USA
[2] Brain Health Coordinating Center, Richmond Veterans Affairs Medical Center, Richmond, VA, USA
[3] Department of Neurosurgery and Neurology, Medical College of Wisconsin, Milwaukee, WI, USA
[4] Psychological and Neurobehavioral Associates, Inc, State College, PA, USA
[5] University of Missouri-Kansas City, Kansas City, MO, USA
[6] University of North Carolina at Chapel Hill, Chapel Hill, NC, USA
[7] Division of Sports Medicine, Boston Children's Hospital, Boston, MA, USA
[8] The Micheli Center for Sports Injury Prevention, Waltham, MA, USA
[9] Departments of Emergency Medicine and Pediatrics, Harvard Medical School, Boston, MA, USA
[10] Division of Emergency Medicine, Boston Children's Hospital, Boston, MA, USA
[11] Department of Kinesiology, Michigan State University, East Lansing, MI, USA
[12] Department of Pediatrics, Harvard Medical School, Boston, MA, USA

Corresponding Author:
Landon B. Lempke, PhD, ATC
1223 E. Marshall St., Suite 410, Egyptian Building, Richmond, VA 23219
E: landon.lempke@vcuhealth.org

Supplementary Table 1. Strongest Dominance Criteria Observed Across TestMyBrain Cognitive Subtests in Former NFL Players (n=295).

DSM

Predictor	Dominance Type	Comparator
AFE	was generally dominated by	LFE
AFE	was completely dominated by	PFCD
AFE	conditionally dominated	LCHx
AFE	was completely dominated by	Age
AFE	generally dominated	HE
AFE	was generally dominated by	Race
LFE	was completely dominated by	PFCD
LFE	generally dominated	LCHx
LFE	was completely dominated by	Age
LFE	generally dominated	HE
LFE	generally dominated	Race
PFCD	completely dominated	LCHx
PFCD	was completely dominated by	Age
PFCD	completely dominated	HE
PFCD	generally dominated	Race
LCHx	was completely dominated by	Age
LCHx	was completely dominated by	HE
LCHx	was completely dominated by	Race
Age	completely dominated	HE
Age	completely dominated	Race
HE	was generally dominated by	Race

Grad CPT

Predictor	Dominance Type	Comparator
AFE	was completely dominated by	LFE
AFE	was generally dominated by	PFCD
AFE	was generally dominated by	LCHx
AFE	was completely dominated by	Age
AFE	was completely dominated by	HE
AFE	was completely dominated by	Race
LFE	generally dominated	PFCD
LFE	generally dominated	LCHx
LFE	was completely dominated by	Age
LFE	was completely dominated by	HE
LFE	was completely dominated by	Race
PFCD	conditionally dominated	LCHx
PFCD	was completely dominated by	Age
PFCD	was completely dominated by	HE
PFCD	was completely dominated by	Race
LCHx	was completely dominated by	Age
LCHx	was completely dominated by	HE
LCHx	was completely dominated by	Race
Age	completely dominated	HE
Age	conditionally dominated	Race
HE	was completely dominated by	Race

Trails A

Predictor	Dominance Type	Comparator
AFE	generally dominated	LFE
AFE	generally dominated	PFCD
AFE	generally dominated	LCHx
AFE	was completely dominated by	Age
AFE	was generally dominated by	HE
AFE	was completely dominated by	Race
LFE	was conditionally dominated by	PFCD
LFE	was generally dominated by	LCHx
LFE	was completely dominated by	Age
LFE	was completely dominated by	HE
LFE	was completely dominated by	Race
PFCD	generally dominated	LCHx
PFCD	was completely dominated by	Age
PFCD	was conditionally dominated by	HE
PFCD	was completely dominated by	Race
LCHx	was completely dominated by	Age
LCHx	was conditionally dominated by	HE
LCHx	was completely dominated by	Race
Age	completely dominated	HE
Age	completely dominated	Race
HE	was completely dominated by	Race

Trails B

Predictor	Dominance Type	Comparator
AFE	conditionally dominated	LFE
AFE	was generally dominated by	PFCD
AFE	conditionally dominated	LCHx
AFE	was completely dominated by	Age
AFE	was conditionally dominated by	HE
AFE	was completely dominated by	Race
LFE	was conditionally dominated by	PFCD
LFE	was generally dominated by	LCHx
LFE	was completely dominated by	Age
LFE	was completely dominated by	HE
LFE	was completely dominated by	Race
PFCD	generally dominated	LCHx
PFCD	was completely dominated by	Age
PFCD	was conditionally dominated by	HE
PFCD	was completely dominated by	Race
LCHx	was completely dominated by	Age
LCHx	was completely dominated by	HE
LCHx	was completely dominated by	Race
Age	completely dominated	HE
Age	completely dominated	Race
HE	was completely dominated by	Race

Verbal PA

Predictor	Dominance Type	Comparator
AFE	was completely dominated by	LFE
AFE	was completely dominated by	PFCD
AFE	was generally dominated by	LCHx
AFE	was completely dominated by	Age
AFE	was completely dominated by	HE
AFE	was completely dominated by	Race
LFE	generally dominated	PFCD
LFE	conditionally dominated	LCHx
LFE	was completely dominated by	Age
LFE	was completely dominated by	HE
LFE	was completely dominated by	Race
PFCD	completely dominated	LCHx
PFCD	was completely dominated by	Age
PFCD	was completely dominated by	HE
PFCD	was completely dominated by	Race
LCHx	was completely dominated by	Age
LCHx	was completely dominated by	HE
LCHx	was completely dominated by	Race
Age	generally dominated	HE
Age	conditionally dominated	Race
HE	completely dominated	Race

Visual PA

Predictor	Dominance Type	Comparator
AFE	was generally dominated by	LFE
AFE	was generally dominated by	PFCD
AFE	generally dominated	LCHx
AFE	was completely dominated by	Age
AFE	was completely dominated by	HE
AFE	was completely dominated by	Race
LFE	was generally dominated by	PFCD
LFE	conditionally dominated	LCHx
LFE	was completely dominated by	Age
LFE	was completely dominated by	HE
LFE	was completely dominated by	Race
PFCD	generally dominated	LCHx
PFCD	was completely dominated by	Age
PFCD	was completely dominated by	HE
PFCD	was completely dominated by	Race
LCHx	was completely dominated by	Age
LCHx	was completely dominated by	HE
LCHx	was completely dominated by	Race
Age	generally dominated	HE
Age	was completely dominated by	Race
HE	was completely dominated by	Race

Vocabulary

Predictor	Dominance Type	Comparator
AFE	was conditionally dominated by	LFE
AFE	was completely dominated by	PFCD
AFE	was generally dominated by	LCHx
AFE	was completely dominated by	Age
AFE	was completely dominated by	HE
AFE	was completely dominated by	Race
LFE	was completely dominated by	PFCD
LFE	was generally dominated by	LCHx
LFE	was completely dominated by	Age
LFE	was completely dominated by	HE
LFE	was completely dominated by	Race
PFCD	conditionally dominated	LCHx
PFCD	was generally dominated by	Age
PFCD	was completely dominated by	HE
PFCD	was completely dominated by	Race
LCHx	was conditionally dominated by	Age
LCHx	was completely dominated by	HE
LCHx	was completely dominated by	Race
Age	was completely dominated by	HE
Age	was completely dominated by	Race
HE	was completely dominated by	Race

Supplementary Table 1 Legend. Dominance Type indicates the type of dominance (general, conditional, complete) with directionality for the predictor relative to the comparator. Only highest dominance criteria reported as lower criteria inherently met if present (i.e., complete dominance also indicates conditional and general dominance present). Abbreviations: Digit Symbol Matching= DSM; Gradual Onset Continuous Performance Test= Grad CPT; Trail Making Test part A= TMT-A; Trail Making Test part B= TMT-B; Verbal Paired Associates Memory= Verbal PA; Visual Paired Associates Memory= Visual PA; AFE= Age of First Exposure to Football; LFE= Lifetime Football Exposure; PFCD = Professional Football Career Duration (years); LCHx = Lifetime Concussion History (total self-reported); HE = Highest Education.

Supplementary Table 2. Dominance Analyses with Bootstrapping across Cognitive Subtests.

Dominance analysis with 1,000 Bootstrap replications across all seven TestMyBrain cognitive subtests. The Dominance column specifies the level of dominance being tested (general, conditional, complete) where general dominance reflects average additional R^2 across all subset models, conditional dominance considers subsets of the same size, and complete dominance requires *i* to dominate *j* in every relevant subset. Each row compares predictors *i* and *j*. *Dij* is the proportion of bootstrap samples in which predictor *i* dominates predictor *j*. *mDij* is the mean dominance value across bootstraps and *SE.Dij* is its standard error. *Pij* is the proportion of samples where *i* does not dominate *j* (*Dij* < 0.5), and *Pji* is the proportion where *j* does not dominate *i*. *Pnoij* indicates the proportion of samples where neither predictor dominates the other. *Rep* is the proportion of bootstrap samples that replicate the observed dominance pattern of *i* over *j*, indicating consistency across samples.

		**DSM**							
**Dominance**	**i**	**j**	**Dij**	**mDij**	**SE.Dij**	**Pij**	**Pji**	**Pnoij**	**Rep**
Complete	Age of First Exposure	Lifetime Football Exposure	0.50	0.49	0.16	0.04	0.06	0.90	0.90
Complete	Age of First Exposure	Professional Career Duration	0.00	0.29	0.25	0.00	0.42	0.58	0.42
Complete	Age of First Exposure	Lifetime Concussion History	0.50	0.58	0.19	0.17	0.01	0.83	0.83
Complete	Age of First Exposure	Age	0.00	0.00	0.00	0.00	1.00	0.00	1.00
Complete	Age of First Exposure	Highest Education	0.50	0.43	0.20	0.02	0.16	0.82	0.82
Complete	Age of First Exposure	Race	0.50	0.48	0.11	0.00	0.05	0.95	0.95
Complete	Lifetime Football Exposure	Professional Career Duration	0.00	0.19	0.24	0.00	0.62	0.38	0.62
Complete	Lifetime Football Exposure	Lifetime Concussion History	0.50	0.51	0.10	0.03	0.01	0.96	0.96
Complete	Lifetime Football Exposure	Age	0.00	0.00	0.00	0.00	1.00	0.00	1.00
Complete	Lifetime Football Exposure	Highest Education	0.50	0.46	0.14	0.00	0.09	0.91	0.91
Complete	Lifetime Football Exposure	Race	0.50	0.49	0.08	0.00	0.03	0.97	0.97
Complete	Professional Career Duration	Lifetime Concussion History	1.00	0.83	0.24	0.67	0.00	0.33	0.67
Complete	Professional Career Duration	Age	0.00	0.00	0.00	0.00	1.00	0.00	1.00
Complete	Professional Career Duration	Highest Education	1.00	0.64	0.23	0.29	0.01	0.70	0.29
Complete	Professional Career Duration	Race	0.50	0.55	0.14	0.09	0.00	0.91	0.91
Complete	Lifetime Concussion History	Age	0.00	0.00	0.00	0.00	1.00	0.00	1.00
Complete	Lifetime Concussion History	Highest Education	0.00	0.21	0.26	0.01	0.60	0.38	0.60
Complete	Lifetime Concussion History	Race	0.00	0.24	0.25	0.00	0.52	0.48	0.52
Complete	Age	Highest Education	1.00	1.00	0.00	1.00	0.00	0.00	1.00
Complete	Age	Race	1.00	1.00	0.00	1.00	0.00	0.00	1.00
Complete	Highest Education	Race	0.50	0.51	0.20	0.09	0.07	0.84	0.84
Conditional	Age of First Exposure	Lifetime Football Exposure	0.50	0.42	0.25	0.06	0.22	0.72	0.72
Conditional	Age of First Exposure	Professional Career Duration	0.00	0.16	0.24	0.00	0.68	0.31	0.68
Conditional	Age of First Exposure	Lifetime Concussion History	1.00	0.73	0.28	0.49	0.04	0.48	0.49
Conditional	Age of First Exposure	Age	0.00	0.00	0.00	0.00	1.00	0.00	1.00
Conditional	Age of First Exposure	Highest Education	0.50	0.40	0.31	0.12	0.32	0.56	0.56
Conditional	Age of First Exposure	Race	0.50	0.41	0.23	0.04	0.22	0.75	0.75
Conditional	Lifetime Football Exposure	Professional Career Duration	0.00	0.13	0.23	0.01	0.76	0.24	0.76

Conditional	Lifetime Football Exposure	Lifetime Concussion History	0.50	0.61	0.22	0.22	0.01	0.77	0.77
Conditional	Lifetime Football Exposure	Age	0.00	0.00	0.00	0.00	1.00	0.00	1.00
Conditional	Lifetime Football Exposure	Highest Education	0.50	0.44	0.19	0.02	0.14	0.84	0.84
Conditional	Lifetime Football Exposure	Race	0.50	0.47	0.13	0.00	0.07	0.93	0.93
Conditional	Professional Career Duration	Lifetime Concussion History	1.00	0.90	0.20	0.79	0.00	0.21	0.79
Conditional	Professional Career Duration	Age	0.00	0.00	0.00	0.00	1.00	0.00	1.00
Conditional	Professional Career Duration	Highest Education	1.00	0.73	0.26	0.47	0.01	0.52	0.47
Conditional	Professional Career Duration	Race	0.50	0.60	0.20	0.19	0.00	0.80	0.80
Conditional	Lifetime Concussion History	Age	0.00	0.00	0.00	0.00	1.00	0.00	1.00
Conditional	Lifetime Concussion History	Highest Education	0.00	0.14	0.26	0.04	0.77	0.19	0.77
Conditional	Lifetime Concussion History	Race	0.00	0.12	0.24	0.02	0.79	0.19	0.79
Conditional	Age	Highest Education	1.00	1.00	0.00	1.00	0.00	0.00	1.00
Conditional	Age	Race	1.00	1.00	0.00	1.00	0.00	0.00	1.00
Conditional	Highest Education	Race	0.50	0.46	0.32	0.17	0.25	0.58	0.58
General	Age of First Exposure	Lifetime Football Exposure	0.00	0.29	0.45	0.29	0.71	0.00	0.71
General	Age of First Exposure	Professional Career Duration	0.00	0.02	0.14	0.02	0.98	0.00	0.98
General	Age of First Exposure	Lifetime Concussion History	1.00	0.87	0.34	0.87	0.13	0.00	0.87
General	Age of First Exposure	Age	0.00	0.00	0.00	0.00	1.00	0.00	1.00
General	Age of First Exposure	Highest Education	1.00	0.39	0.49	0.39	0.61	0.00	0.39
General	Age of First Exposure	Race	0.00	0.26	0.44	0.26	0.74	0.00	0.74
General	Lifetime Football Exposure	Professional Career Duration	0.00	0.06	0.24	0.06	0.94	0.00	0.94
General	Lifetime Football Exposure	Lifetime Concussion History	1.00	0.94	0.24	0.94	0.06	0.00	0.94
General	Lifetime Football Exposure	Age	0.00	0.00	0.00	0.00	1.00	0.00	1.00
General	Lifetime Football Exposure	Highest Education	1.00	0.56	0.50	0.56	0.44	0.00	0.56
General	Lifetime Football Exposure	Race	1.00	0.45	0.50	0.45	0.55	0.00	0.45
General	Professional Career Duration	Lifetime Concussion History	1.00	1.00	0.03	1.00	0.00	0.00	1.00
General	Professional Career Duration	Age	0.00	0.00	0.00	0.00	1.00	0.00	1.00
General	Professional Career Duration	Highest Education	1.00	0.95	0.22	0.95	0.05	0.00	0.95
General	Professional Career Duration	Race	1.00	0.93	0.26	0.93	0.08	0.00	0.93
General	Lifetime Concussion History	Age	0.00	0.00	0.00	0.00	1.00	0.00	1.00
General	Lifetime Concussion History	Highest Education	0.00	0.09	0.29	0.09	0.91	0.00	0.91
General	Lifetime Concussion History	Race	0.00	0.06	0.23	0.06	0.94	0.00	0.94
General	Age	Highest Education	1.00	1.00	0.00	1.00	0.00	0.00	1.00
General	Age	Race	1.00	1.00	0.00	1.00	0.00	0.00	1.00
General	Highest Education	Race	0.00	0.39	0.49	0.39	0.61	0.00	0.61

Grad CPT

**Dominance**	**i**	**j**	**Dij**	**mDij**	**SE.Dij**	**Pij**	**Pji**	**Pnoij**	**Rep**
Complete	Age of First Exposure	Lifetime Football Exposure	0.00	0.30	0.28	0.03	0.44	0.53	0.44
Complete	Age of First Exposure	Professional Career Duration	0.50	0.43	0.19	0.01	0.15	0.83	0.83
Complete	Age of First Exposure	Lifetime Concussion History	0.50	0.45	0.20	0.03	0.13	0.84	0.84

Complete	Age of First Exposure	Age	0.00	0.05	0.14	0.00	0.91	0.09	0.91
Complete	Age of First Exposure	Highest Education	0.00	0.09	0.20	0.00	0.82	0.18	0.82
Complete	Age of First Exposure	Race	0.00	0.07	0.17	0.00	0.86	0.14	0.86
Complete	Lifetime Football Exposure	Professional Career Duration	0.50	0.50	0.13	0.03	0.03	0.93	0.93
Complete	Lifetime Football Exposure	Lifetime Concussion History	0.50	0.55	0.20	0.13	0.04	0.83	0.83
Complete	Lifetime Football Exposure	Age	0.00	0.15	0.23	0.01	0.71	0.28	0.71
Complete	Lifetime Football Exposure	Highest Education	0.00	0.21	0.26	0.01	0.59	0.40	0.59
Complete	Lifetime Football Exposure	Race	0.00	0.15	0.23	0.00	0.71	0.29	0.71
Complete	Professional Career Duration	Lifetime Concussion History	0.50	0.53	0.17	0.09	0.03	0.88	0.88
Complete	Professional Career Duration	Age	0.00	0.09	0.20	0.01	0.83	0.16	0.83
Complete	Professional Career Duration	Highest Education	0.00	0.18	0.24	0.00	0.65	0.34	0.65
Complete	Professional Career Duration	Race	0.00	0.14	0.23	0.00	0.73	0.27	0.73
Complete	Lifetime Concussion History	Age	0.00	0.06	0.17	0.00	0.89	0.11	0.89
Complete	Lifetime Concussion History	Highest Education	0.00	0.08	0.19	0.01	0.86	0.14	0.86
Complete	Lifetime Concussion History	Race	0.00	0.03	0.13	0.00	0.93	0.07	0.93
Complete	Age	Highest Education	1.00	0.56	0.38	0.36	0.23	0.41	0.36
Complete	Age	Race	0.50	0.49	0.36	0.25	0.28	0.47	0.47
Complete	Highest Education	Race	0.00	0.42	0.42	0.28	0.44	0.29	0.44
Conditional	Age of First Exposure	Lifetime Football Exposure	0.00	0.22	0.34	0.10	0.66	0.24	0.66
Conditional	Age of First Exposure	Professional Career Duration	0.50	0.39	0.28	0.08	0.30	0.63	0.63
Conditional	Age of First Exposure	Lifetime Concussion History	0.50	0.44	0.32	0.15	0.27	0.58	0.58
Conditional	Age of First Exposure	Age	0.00	0.02	0.11	0.00	0.95	0.04	0.95
Conditional	Age of First Exposure	Highest Education	0.00	0.06	0.17	0.01	0.89	0.10	0.89
Conditional	Age of First Exposure	Race	0.00	0.04	0.14	0.01	0.93	0.06	0.93
Conditional	Lifetime Football Exposure	Professional Career Duration	0.50	0.55	0.29	0.22	0.12	0.66	0.66
Conditional	Lifetime Football Exposure	Lifetime Concussion History	0.50	0.64	0.31	0.37	0.09	0.54	0.54
Conditional	Lifetime Football Exposure	Age	0.00	0.08	0.20	0.01	0.85	0.14	0.85
Conditional	Lifetime Football Exposure	Highest Education	0.00	0.14	0.25	0.03	0.75	0.23	0.75
Conditional	Lifetime Football Exposure	Race	0.00	0.10	0.21	0.01	0.81	0.18	0.81
Conditional	Professional Career Duration	Lifetime Concussion History	1.00	0.61	0.36	0.40	0.18	0.42	0.40
Conditional	Professional Career Duration	Age	0.00	0.06	0.20	0.03	0.91	0.07	0.91
Conditional	Professional Career Duration	Highest Education	0.00	0.10	0.21	0.01	0.80	0.19	0.80
Conditional	Professional Career Duration	Race	0.00	0.08	0.19	0.01	0.85	0.15	0.85
Conditional	Lifetime Concussion History	Age	0.00	0.03	0.14	0.01	0.96	0.03	0.96
Conditional	Lifetime Concussion History	Highest Education	0.00	0.04	0.16	0.01	0.93	0.06	0.93
Conditional	Lifetime Concussion History	Race	0.00	0.02	0.11	0.00	0.96	0.04	0.96
Conditional	Age	Highest Education	1.00	0.58	0.44	0.48	0.32	0.20	0.48
Conditional	Age	Race	1.00	0.52	0.44	0.41	0.38	0.21	0.41
Conditional	Highest Education	Race	0.00	0.42	0.45	0.34	0.49	0.17	0.49
General	Age of First Exposure	Lifetime Football Exposure	0.00	0.15	0.36	0.15	0.85	0.00	0.85

General	Age of First Exposure	Professional Career Duration	0.00	0.20	0.40	0.20	0.80	0.00	0.80
General	Age of First Exposure	Lifetime Concussion History	0.00	0.32	0.47	0.32	0.68	0.00	0.68
General	Age of First Exposure	Age	0.00	0.01	0.10	0.01	0.99	0.00	0.99
General	Age of First Exposure	Highest Education	0.00	0.03	0.18	0.03	0.97	0.00	0.97
General	Age of First Exposure	Race	0.00	0.01	0.11	0.01	0.99	0.00	0.99
General	Lifetime Football Exposure	Professional Career Duration	1.00	0.59	0.49	0.59	0.41	0.00	0.59
General	Lifetime Football Exposure	Lifetime Concussion History	1.00	0.79	0.41	0.79	0.21	0.00	0.79
General	Lifetime Football Exposure	Age	0.00	0.07	0.25	0.07	0.94	0.00	0.94
General	Lifetime Football Exposure	Highest Education	0.00	0.13	0.33	0.13	0.87	0.00	0.87
General	Lifetime Football Exposure	Race	0.00	0.07	0.26	0.07	0.93	0.00	0.93
General	Professional Career Duration	Lifetime Concussion History	1.00	0.69	0.46	0.69	0.31	0.00	0.69
General	Professional Career Duration	Age	0.00	0.05	0.21	0.05	0.95	0.00	0.95
General	Professional Career Duration	Highest Education	0.00	0.06	0.24	0.06	0.94	0.00	0.94
General	Professional Career Duration	Race	0.00	0.05	0.21	0.05	0.96	0.00	0.96
General	Lifetime Concussion History	Age	0.00	0.02	0.14	0.02	0.98	0.00	0.98
General	Lifetime Concussion History	Highest Education	0.00	0.03	0.17	0.03	0.97	0.00	0.97
General	Lifetime Concussion History	Race	0.00	0.01	0.11	0.01	0.99	0.00	0.99
General	Age	Highest Education	1.00	0.58	0.49	0.58	0.42	0.00	0.58
General	Age	Race	1.00	0.52	0.50	0.52	0.48	0.00	0.52
General	Highest Education	Race	0.00	0.42	0.49	0.42	0.58	0.00	0.58

Trails A

**Dominance**	**i**	**j**	**Dij**	**mDij**	**SE.Dij**	**Pij**	**Pji**	**Pnoij**	**Rep**
Complete	Age of First Exposure	Lifetime Football Exposure	0.50	0.54	0.15	0.10	0.01	0.90	0.90
Complete	Age of First Exposure	Professional Career Duration	0.50	0.51	0.10	0.03	0.01	0.96	0.96
Complete	Age of First Exposure	Lifetime Concussion History	0.50	0.53	0.15	0.07	0.02	0.90	0.90
Complete	Age of First Exposure	Age	0.00	0.00	0.02	0.00	1.00	0.00	1.00
Complete	Age of First Exposure	Highest Education	0.50	0.33	0.24	0.01	0.35	0.65	0.65
Complete	Age of First Exposure	Race	0.00	0.04	0.13	0.00	0.92	0.08	0.92
Complete	Lifetime Football Exposure	Professional Career Duration	0.50	0.50	0.08	0.01	0.01	0.97	0.97
Complete	Lifetime Football Exposure	Lifetime Concussion History	0.50	0.48	0.13	0.01	0.05	0.93	0.93
Complete	Lifetime Football Exposure	Age	0.00	0.00	0.00	0.00	1.00	0.00	1.00
Complete	Lifetime Football Exposure	Highest Education	0.00	0.28	0.25	0.00	0.44	0.56	0.44
Complete	Lifetime Football Exposure	Race	0.00	0.04	0.14	0.00	0.91	0.09	0.91
Complete	Professional Career Duration	Lifetime Concussion History	0.50	0.51	0.15	0.06	0.04	0.91	0.91
Complete	Professional Career Duration	Age	0.00	0.00	0.00	0.00	1.00	0.00	1.00
Complete	Professional Career Duration	Highest Education	0.50	0.28	0.25	0.00	0.43	0.57	0.57
Complete	Professional Career Duration	Race	0.00	0.05	0.15	0.00	0.90	0.11	0.90
Complete	Lifetime Concussion History	Age	0.00	0.00	0.00	0.00	1.00	0.00	1.00
Complete	Lifetime Concussion History	Highest Education	0.50	0.18	0.25	0.01	0.65	0.34	0.34
Complete	Lifetime Concussion History	Race	0.00	0.02	0.09	0.00	0.97	0.03	0.97

Complete	Age	Highest Education	1.00	1.00	0.04	0.99	0.00	0.01	0.99
Complete	Age	Race	1.00	0.97	0.13	0.94	0.00	0.05	0.94
Complete	Highest Education	Race	0.00	0.14	0.25	0.02	0.74	0.24	0.74
Conditional	Age of First Exposure	Lifetime Football Exposure	0.50	0.58	0.26	0.22	0.07	0.71	0.71
Conditional	Age of First Exposure	Professional Career Duration	0.50	0.49	0.24	0.10	0.13	0.77	0.77
Conditional	Age of First Exposure	Lifetime Concussion History	0.50	0.61	0.31	0.33	0.11	0.56	0.56
Conditional	Age of First Exposure	Age	0.00	0.00	0.00	0.00	1.00	0.00	1.00
Conditional	Age of First Exposure	Highest Education	0.50	0.24	0.27	0.02	0.54	0.45	0.45
Conditional	Age of First Exposure	Race	0.00	0.01	0.07	0.00	0.98	0.02	0.98
Conditional	Lifetime Football Exposure	Professional Career Duration	0.00	0.42	0.27	0.08	0.25	0.67	0.25
Conditional	Lifetime Football Exposure	Lifetime Concussion History	0.50	0.50	0.32	0.21	0.21	0.59	0.59
Conditional	Lifetime Football Exposure	Age	0.00	0.00	0.00	0.00	1.00	0.00	1.00
Conditional	Lifetime Football Exposure	Highest Education	0.00	0.13	0.23	0.01	0.74	0.25	0.74
Conditional	Lifetime Football Exposure	Race	0.00	0.01	0.09	0.00	0.97	0.03	0.97
Conditional	Professional Career Duration	Lifetime Concussion History	0.50	0.61	0.30	0.31	0.10	0.59	0.59
Conditional	Professional Career Duration	Age	0.00	0.00	0.00	0.00	1.00	0.00	1.00
Conditional	Professional Career Duration	Highest Education	0.00	0.20	0.26	0.02	0.61	0.38	0.61
Conditional	Professional Career Duration	Race	0.00	0.03	0.11	0.00	0.95	0.05	0.95
Conditional	Lifetime Concussion History	Age	0.00	0.00	0.00	0.00	1.00	0.00	1.00
Conditional	Lifetime Concussion History	Highest Education	0.00	0.12	0.27	0.06	0.82	0.12	0.82
Conditional	Lifetime Concussion History	Race	0.00	0.01	0.07	0.00	0.98	0.02	0.98
Conditional	Age	Highest Education	1.00	1.00	0.03	1.00	0.00	0.00	1.00
Conditional	Age	Race	1.00	0.99	0.09	0.98	0.01	0.02	0.98
Conditional	Highest Education	Race	0.00	0.10	0.24	0.04	0.84	0.12	0.84
General	Age of First Exposure	Lifetime Football Exposure	1.00	0.67	0.47	0.67	0.33	0.00	0.67
General	Age of First Exposure	Professional Career Duration	1.00	0.55	0.50	0.55	0.45	0.00	0.55
General	Age of First Exposure	Lifetime Concussion History	1.00	0.73	0.44	0.73	0.27	0.00	0.73
General	Age of First Exposure	Age	0.00	0.00	0.00	0.00	1.00	0.00	1.00
General	Age of First Exposure	Highest Education	0.00	0.17	0.38	0.17	0.83	0.00	0.83
General	Age of First Exposure	Race	0.00	0.00	0.05	0.00	1.00	0.00	1.00
General	Lifetime Football Exposure	Professional Career Duration	0.00	0.33	0.47	0.33	0.67	0.00	0.67
General	Lifetime Football Exposure	Lifetime Concussion History	0.00	0.62	0.49	0.62	0.38	0.00	0.38
General	Lifetime Football Exposure	Age	0.00	0.00	0.00	0.00	1.00	0.00	1.00
General	Lifetime Football Exposure	Highest Education	0.00	0.08	0.27	0.08	0.92	0.00	0.92
General	Lifetime Football Exposure	Race	0.00	0.00	0.03	0.00	1.00	0.00	1.00
General	Professional Career Duration	Lifetime Concussion History	1.00	0.72	0.45	0.72	0.28	0.00	0.72
General	Professional Career Duration	Age	0.00	0.00	0.00	0.00	1.00	0.00	1.00
General	Professional Career Duration	Highest Education	0.00	0.13	0.33	0.13	0.87	0.00	0.87
General	Professional Career Duration	Race	0.00	0.00	0.05	0.00	1.00	0.00	1.00
General	Lifetime Concussion History	Age	0.00	0.00	0.00	0.00	1.00	0.00	1.00

General	Lifetime Concussion History	Highest Education	0.00	0.09	0.29	0.09	0.91	0.00	0.91
General	Lifetime Concussion History	Race	0.00	0.00	0.06	0.00	1.00	0.00	1.00
General	Age	Highest Education	1.00	1.00	0.03	1.00	0.00	0.00	1.00
General	Age	Race	1.00	0.99	0.10	0.99	0.01	0.00	0.99
General	Highest Education	Race	0.00	0.08	0.27	0.08	0.92	0.00	0.92

Trails B

**Dominance**	**i**	**j**	**Dij**	**mDij**	**SE.Dij**	**Pij**	**Pji**	**Pnoij**	**Rep**
Complete	Age of First Exposure	Lifetime Football Exposure	0.50	0.60	0.20	0.20	0.01	0.80	0.80
Complete	Age of First Exposure	Professional Career Duration	0.50	0.51	0.14	0.04	0.03	0.93	0.93
Complete	Age of First Exposure	Lifetime Concussion History	0.50	0.53	0.20	0.11	0.05	0.84	0.84
Complete	Age of First Exposure	Age	0.00	0.00	0.02	0.00	1.00	0.00	1.00
Complete	Age of First Exposure	Highest Education	0.50	0.31	0.24	0.00	0.39	0.61	0.61
Complete	Age of First Exposure	Race	0.00	0.08	0.18	0.00	0.84	0.16	0.84
Complete	Lifetime Football Exposure	Professional Career Duration	0.50	0.46	0.14	0.00	0.08	0.92	0.92
Complete	Lifetime Football Exposure	Lifetime Concussion History	0.50	0.44	0.17	0.00	0.12	0.88	0.88
Complete	Lifetime Football Exposure	Age	0.00	0.00	0.02	0.00	1.00	0.00	1.00
Complete	Lifetime Football Exposure	Highest Education	0.00	0.22	0.25	0.00	0.57	0.43	0.57
Complete	Lifetime Football Exposure	Race	0.00	0.08	0.19	0.00	0.83	0.17	0.83
Complete	Professional Career Duration	Lifetime Concussion History	0.50	0.50	0.16	0.05	0.05	0.90	0.90
Complete	Professional Career Duration	Age	0.00	0.00	0.02	0.00	1.00	0.00	1.00
Complete	Professional Career Duration	Highest Education	0.50	0.26	0.25	0.00	0.48	0.52	0.52
Complete	Professional Career Duration	Race	0.00	0.09	0.19	0.00	0.82	0.18	0.82
Complete	Lifetime Concussion History	Age	0.00	0.00	0.00	0.00	1.00	0.00	1.00
Complete	Lifetime Concussion History	Highest Education	0.00	0.17	0.25	0.01	0.67	0.32	0.67
Complete	Lifetime Concussion History	Race	0.00	0.03	0.13	0.00	0.93	0.07	0.93
Complete	Age	Highest Education	1.00	0.98	0.11	0.95	0.00	0.05	0.95
Complete	Age	Race	1.00	0.87	0.24	0.77	0.02	0.21	0.77
Complete	Highest Education	Race	0.00	0.16	0.25	0.02	0.70	0.29	0.70
Conditional	Age of First Exposure	Lifetime Football Exposure	1.00	0.68	0.30	0.43	0.07	0.50	0.43
Conditional	Age of First Exposure	Professional Career Duration	0.50	0.56	0.30	0.24	0.12	0.63	0.63
Conditional	Age of First Exposure	Lifetime Concussion History	1.00	0.62	0.37	0.43	0.18	0.38	0.43
Conditional	Age of First Exposure	Age	0.00	0.00	0.00	0.00	1.00	0.00	1.00
Conditional	Age of First Exposure	Highest Education	0.00	0.16	0.27	0.04	0.72	0.25	0.72
Conditional	Age of First Exposure	Race	0.00	0.03	0.12	0.00	0.94	0.06	0.94
Conditional	Lifetime Football Exposure	Professional Career Duration	0.00	0.38	0.28	0.06	0.30	0.64	0.30
Conditional	Lifetime Football Exposure	Lifetime Concussion History	0.50	0.42	0.30	0.11	0.27	0.62	0.62
Conditional	Lifetime Football Exposure	Age	0.00	0.00	0.00	0.00	1.00	0.00	1.00
Conditional	Lifetime Football Exposure	Highest Education	0.00	0.09	0.19	0.00	0.82	0.18	0.82
Conditional	Lifetime Football Exposure	Race	0.00	0.02	0.09	0.00	0.96	0.04	0.96
Conditional	Professional Career Duration	Lifetime Concussion History	0.50	0.56	0.30	0.25	0.13	0.62	0.62

Conditional	Professional Career Duration	Age	0.00	0.00	0.03	0.00	1.00	0.00	1.00
Conditional	Professional Career Duration	Highest Education	0.00	0.15	0.24	0.01	0.70	0.29	0.70
Conditional	Professional Career Duration	Race	0.00	0.05	0.15	0.00	0.91	0.09	0.91
Conditional	Lifetime Concussion History	Age	0.00	0.00	0.00	0.00	1.00	0.00	1.00
Conditional	Lifetime Concussion History	Highest Education	0.00	0.10	0.25	0.05	0.85	0.10	0.85
Conditional	Lifetime Concussion History	Race	0.00	0.01	0.09	0.00	0.97	0.02	0.97
Conditional	Age	Highest Education	1.00	0.99	0.06	0.99	0.00	0.01	0.99
Conditional	Age	Race	1.00	0.92	0.24	0.89	0.05	0.06	0.89
Conditional	Highest Education	Race	0.00	0.12	0.26	0.04	0.81	0.15	0.81
General	Age of First Exposure	Lifetime Football Exposure	1.00	0.77	0.42	0.77	0.24	0.00	0.77
General	Age of First Exposure	Professional Career Duration	0.00	0.48	0.50	0.48	0.52	0.00	0.52
General	Age of First Exposure	Lifetime Concussion History	1.00	0.64	0.48	0.64	0.36	0.00	0.64
General	Age of First Exposure	Age	0.00	0.00	0.00	0.00	1.00	0.00	1.00
General	Age of First Exposure	Highest Education	0.00	0.09	0.29	0.09	0.91	0.00	0.91
General	Age of First Exposure	Race	0.00	0.00	0.05	0.00	1.00	0.00	1.00
General	Lifetime Football Exposure	Professional Career Duration	0.00	0.26	0.44	0.26	0.74	0.00	0.74
General	Lifetime Football Exposure	Lifetime Concussion History	0.00	0.49	0.50	0.49	0.51	0.00	0.51
General	Lifetime Football Exposure	Age	0.00	0.00	0.00	0.00	1.00	0.00	1.00
General	Lifetime Football Exposure	Highest Education	0.00	0.03	0.16	0.03	0.97	0.00	0.97
General	Lifetime Football Exposure	Race	0.00	0.00	0.05	0.00	1.00	0.00	1.00
General	Professional Career Duration	Lifetime Concussion History	1.00	0.66	0.48	0.66	0.34	0.00	0.66
General	Professional Career Duration	Age	0.00	0.00	0.03	0.00	1.00	0.00	1.00
General	Professional Career Duration	Highest Education	0.00	0.08	0.27	0.08	0.92	0.00	0.92
General	Professional Career Duration	Race	0.00	0.01	0.08	0.01	0.99	0.00	0.99
General	Lifetime Concussion History	Age	0.00	0.00	0.00	0.00	1.00	0.00	1.00
General	Lifetime Concussion History	Highest Education	0.00	0.07	0.26	0.07	0.93	0.00	0.93
General	Lifetime Concussion History	Race	0.00	0.00	0.06	0.00	1.00	0.00	1.00
General	Age	Highest Education	1.00	1.00	0.06	1.00	0.00	0.00	1.00
General	Age	Race	1.00	0.93	0.26	0.93	0.07	0.00	0.93
General	Highest Education	Race	0.00	0.08	0.27	0.08	0.92	0.00	0.92

Verbal PA

**Dominance**	**i**	**j**	**Dij**	**mDij**	**SE.Dij**	**Pij**	**Pji**	**Pnoij**	**Rep**
Complete	Age of First Exposure	Lifetime Football Exposure	0.00	0.26	0.25	0.01	0.48	0.52	0.48
Complete	Age of First Exposure	Professional Career Duration	0.00	0.34	0.24	0.00	0.33	0.67	0.33
Complete	Age of First Exposure	Lifetime Concussion History	0.50	0.44	0.18	0.02	0.13	0.85	0.85
Complete	Age of First Exposure	Age	0.00	0.06	0.16	0.00	0.89	0.11	0.89
Complete	Age of First Exposure	Highest Education	0.00	0.03	0.12	0.00	0.93	0.07	0.93
Complete	Age of First Exposure	Race	0.00	0.06	0.17	0.00	0.87	0.13	0.87
Complete	Lifetime Football Exposure	Professional Career Duration	0.50	0.50	0.23	0.10	0.11	0.79	0.79
Complete	Lifetime Football Exposure	Lifetime Concussion History	0.50	0.60	0.25	0.24	0.05	0.71	0.71

Complete	Lifetime Football Exposure	Age	0.00	0.13	0.22	0.01	0.75	0.24	0.75
Complete	Lifetime Football Exposure	Highest Education	0.00	0.12	0.21	0.00	0.76	0.24	0.76
Complete	Lifetime Football Exposure	Race	0.00	0.22	0.25	0.00	0.57	0.43	0.57
Complete	Professional Career Duration	Lifetime Concussion History	1.00	0.62	0.26	0.28	0.04	0.68	0.28
Complete	Professional Career Duration	Age	0.00	0.11	0.22	0.01	0.79	0.20	0.79
Complete	Professional Career Duration	Highest Education	0.00	0.11	0.21	0.00	0.78	0.22	0.78
Complete	Professional Career Duration	Race	0.00	0.19	0.25	0.01	0.63	0.36	0.63
Complete	Lifetime Concussion History	Age	0.00	0.05	0.15	0.00	0.91	0.09	0.91
Complete	Lifetime Concussion History	Highest Education	0.00	0.03	0.13	0.00	0.94	0.06	0.94
Complete	Lifetime Concussion History	Race	0.00	0.05	0.16	0.01	0.91	0.09	0.91
Complete	Age	Highest Education	0.50	0.47	0.33	0.18	0.25	0.57	0.57
Complete	Age	Race	0.50	0.59	0.32	0.32	0.13	0.56	0.56
Complete	Highest Education	Race	1.00	0.67	0.36	0.50	0.15	0.35	0.50
Conditional	Age of First Exposure	Lifetime Football Exposure	0.00	0.13	0.27	0.05	0.78	0.17	0.78
Conditional	Age of First Exposure	Professional Career Duration	0.00	0.20	0.26	0.02	0.62	0.37	0.62
Conditional	Age of First Exposure	Lifetime Concussion History	0.50	0.41	0.33	0.14	0.32	0.54	0.54
Conditional	Age of First Exposure	Age	0.00	0.01	0.07	0.00	0.98	0.02	0.98
Conditional	Age of First Exposure	Highest Education	0.00	0.01	0.08	0.00	0.97	0.03	0.97
Conditional	Age of First Exposure	Race	0.00	0.02	0.11	0.00	0.95	0.05	0.95
Conditional	Lifetime Football Exposure	Professional Career Duration	0.50	0.52	0.37	0.29	0.25	0.46	0.46
Conditional	Lifetime Football Exposure	Lifetime Concussion History	1.00	0.73	0.37	0.61	0.14	0.25	0.61
Conditional	Lifetime Football Exposure	Age	0.00	0.06	0.19	0.02	0.91	0.07	0.91
Conditional	Lifetime Football Exposure	Highest Education	0.00	0.05	0.18	0.02	0.91	0.07	0.91
Conditional	Lifetime Football Exposure	Race	0.00	0.09	0.23	0.03	0.85	0.12	0.85
Conditional	Professional Career Duration	Lifetime Concussion History	1.00	0.72	0.33	0.53	0.09	0.38	0.53
Conditional	Professional Career Duration	Age	0.00	0.07	0.22	0.03	0.89	0.08	0.89
Conditional	Professional Career Duration	Highest Education	0.00	0.06	0.20	0.02	0.90	0.07	0.90
Conditional	Professional Career Duration	Race	0.00	0.13	0.24	0.02	0.77	0.21	0.77
Conditional	Lifetime Concussion History	Age	0.00	0.01	0.10	0.01	0.98	0.01	0.98
Conditional	Lifetime Concussion History	Highest Education	0.00	0.02	0.11	0.01	0.97	0.02	0.97
Conditional	Lifetime Concussion History	Race	0.00	0.02	0.13	0.01	0.96	0.03	0.96
Conditional	Age	Highest Education	0.50	0.48	0.45	0.39	0.43	0.18	0.18
Conditional	Age	Race	1.00	0.67	0.42	0.59	0.25	0.17	0.59
Conditional	Highest Education	Race	1.00	0.69	0.42	0.62	0.23	0.14	0.62
General	Age of First Exposure	Lifetime Football Exposure	0.00	0.10	0.30	0.10	0.90	0.00	0.90
General	Age of First Exposure	Professional Career Duration	0.00	0.12	0.33	0.12	0.88	0.00	0.88
General	Age of First Exposure	Lifetime Concussion History	0.00	0.42	0.49	0.42	0.58	0.00	0.58
General	Age of First Exposure	Age	0.00	0.00	0.04	0.00	1.00	0.00	1.00
General	Age of First Exposure	Highest Education	0.00	0.00	0.04	0.00	1.00	0.00	1.00
General	Age of First Exposure	Race	0.00	0.01	0.08	0.01	0.99	0.00	0.99

General	Lifetime Football Exposure	Professional Career Duration	1.00	0.50	0.50	0.50	0.51	0.00	0.50
General	Lifetime Football Exposure	Lifetime Concussion History	1.00	0.79	0.41	0.79	0.21	0.00	0.79
General	Lifetime Football Exposure	Age	0.00	0.05	0.22	0.05	0.95	0.00	0.95
General	Lifetime Football Exposure	Highest Education	0.00	0.04	0.19	0.04	0.96	0.00	0.96
General	Lifetime Football Exposure	Race	0.00	0.06	0.23	0.06	0.94	0.00	0.94
General	Professional Career Duration	Lifetime Concussion History	1.00	0.82	0.39	0.82	0.18	0.00	0.82
General	Professional Career Duration	Age	0.00	0.06	0.24	0.06	0.94	0.00	0.94
General	Professional Career Duration	Highest Education	0.00	0.04	0.20	0.04	0.96	0.00	0.96
General	Professional Career Duration	Race	0.00	0.09	0.29	0.09	0.91	0.00	0.91
General	Lifetime Concussion History	Age	0.00	0.01	0.09	0.01	0.99	0.00	0.99
General	Lifetime Concussion History	Highest Education	0.00	0.01	0.12	0.01	0.99	0.00	0.99
General	Lifetime Concussion History	Race	0.00	0.02	0.13	0.02	0.98	0.00	0.98
General	Age	Highest Education	1.00	0.47	0.50	0.47	0.53	0.00	0.47
General	Age	Race	1.00	0.67	0.47	0.67	0.33	0.00	0.67
General	Highest Education	Race	1.00	0.69	0.46	0.69	0.31	0.00	0.69
		**Visual PA**							
**Dominance**	**i**	**j**	**Dij**	**mDij**	**SE.Dij**	**Pij**	**Pji**	**Pnoij**	**Rep**
Complete	Age of First Exposure	Lifetime Football Exposure	0.50	0.54	0.24	0.16	0.07	0.77	0.77
Complete	Age of First Exposure	Professional Career Duration	0.50	0.50	0.22	0.10	0.10	0.81	0.81
Complete	Age of First Exposure	Lifetime Concussion History	0.50	0.51	0.24	0.13	0.11	0.76	0.76
Complete	Age of First Exposure	Age	0.00	0.18	0.24	0.00	0.64	0.35	0.64
Complete	Age of First Exposure	Highest Education	0.00	0.20	0.26	0.01	0.61	0.38	0.61
Complete	Age of First Exposure	Race	0.00	0.00	0.05	0.00	0.99	0.01	0.99
Complete	Lifetime Football Exposure	Professional Career Duration	0.50	0.49	0.18	0.06	0.08	0.87	0.87
Complete	Lifetime Football Exposure	Lifetime Concussion History	0.50	0.46	0.19	0.04	0.11	0.85	0.85
Complete	Lifetime Football Exposure	Age	0.00	0.14	0.22	0.00	0.73	0.27	0.73
Complete	Lifetime Football Exposure	Highest Education	0.00	0.18	0.24	0.00	0.65	0.35	0.65
Complete	Lifetime Football Exposure	Race	0.00	0.00	0.02	0.00	1.00	0.00	1.00
Complete	Professional Career Duration	Lifetime Concussion History	0.50	0.48	0.23	0.09	0.13	0.79	0.79
Complete	Professional Career Duration	Age	0.00	0.14	0.24	0.01	0.73	0.26	0.73
Complete	Professional Career Duration	Highest Education	0.00	0.18	0.25	0.01	0.66	0.33	0.66
Complete	Professional Career Duration	Race	0.00	0.01	0.05	0.00	0.99	0.01	0.99
Complete	Lifetime Concussion History	Age	0.00	0.13	0.23	0.01	0.74	0.24	0.74
Complete	Lifetime Concussion History	Highest Education	0.00	0.12	0.24	0.02	0.77	0.21	0.77
Complete	Lifetime Concussion History	Race	0.00	0.00	0.04	0.00	1.00	0.01	1.00
Complete	Age	Highest Education	0.50	0.58	0.27	0.24	0.08	0.68	0.68
Complete	Age	Race	0.00	0.07	0.21	0.02	0.88	0.10	0.88
Complete	Highest Education	Race	0.00	0.03	0.13	0.01	0.96	0.04	0.96
Conditional	Age of First Exposure	Lifetime Football Exposure	0.50	0.53	0.38	0.32	0.26	0.42	0.42
Conditional	Age of First Exposure	Professional Career Duration	0.50	0.52	0.35	0.26	0.23	0.51	0.51

Conditional	Age of First Exposure	Lifetime Concussion History	0.50	0.51	0.39	0.31	0.29	0.40	0.40
Conditional	Age of First Exposure	Age	0.00	0.09	0.23	0.03	0.86	0.11	0.86
Conditional	Age of First Exposure	Highest Education	0.00	0.10	0.21	0.02	0.82	0.16	0.82
Conditional	Age of First Exposure	Race	0.00	0.00	0.02	0.00	1.00	0.00	1.00
Conditional	Lifetime Football Exposure	Professional Career Duration	0.50	0.52	0.33	0.24	0.21	0.56	0.56
Conditional	Lifetime Football Exposure	Lifetime Concussion History	1.00	0.51	0.37	0.28	0.27	0.45	0.28
Conditional	Lifetime Football Exposure	Age	0.00	0.06	0.17	0.01	0.89	0.11	0.89
Conditional	Lifetime Football Exposure	Highest Education	0.00	0.08	0.19	0.01	0.86	0.13	0.86
Conditional	Lifetime Football Exposure	Race	0.00	0.00	0.00	0.00	1.00	0.00	1.00
Conditional	Professional Career Duration	Lifetime Concussion History	0.50	0.48	0.36	0.24	0.29	0.48	0.48
Conditional	Professional Career Duration	Age	0.00	0.10	0.24	0.03	0.82	0.15	0.82
Conditional	Professional Career Duration	Highest Education	0.00	0.09	0.23	0.03	0.86	0.11	0.86
Conditional	Professional Career Duration	Race	0.00	0.00	0.03	0.00	1.00	0.00	1.00
Conditional	Lifetime Concussion History	Age	0.00	0.08	0.22	0.03	0.87	0.10	0.87
Conditional	Lifetime Concussion History	Highest Education	0.00	0.10	0.25	0.05	0.86	0.10	0.86
Conditional	Lifetime Concussion History	Race	0.00	0.00	0.02	0.00	1.00	0.00	1.00
Conditional	Age	Highest Education	0.50	0.60	0.36	0.38	0.18	0.44	0.44
Conditional	Age	Race	0.00	0.06	0.21	0.04	0.92	0.05	0.92
Conditional	Highest Education	Race	0.00	0.02	0.13	0.01	0.97	0.02	0.97
General	Age of First Exposure	Lifetime Football Exposure	0.00	0.56	0.50	0.56	0.45	0.00	0.45
General	Age of First Exposure	Professional Career Duration	0.00	0.53	0.50	0.53	0.47	0.00	0.47
General	Age of First Exposure	Lifetime Concussion History	1.00	0.56	0.50	0.56	0.44	0.00	0.56
General	Age of First Exposure	Age	0.00	0.06	0.24	0.06	0.94	0.00	0.94
General	Age of First Exposure	Highest Education	0.00	0.06	0.23	0.06	0.94	0.00	0.94
General	Age of First Exposure	Race	0.00	0.00	0.00	0.00	1.00	0.00	1.00
General	Lifetime Football Exposure	Professional Career Duration	0.00	0.51	0.50	0.51	0.50	0.00	0.50
General	Lifetime Football Exposure	Lifetime Concussion History	1.00	0.58	0.49	0.58	0.42	0.00	0.58
General	Lifetime Football Exposure	Age	0.00	0.03	0.17	0.03	0.97	0.00	0.97
General	Lifetime Football Exposure	Highest Education	0.00	0.04	0.18	0.04	0.97	0.00	0.97
General	Lifetime Football Exposure	Race	0.00	0.00	0.00	0.00	1.00	0.00	1.00
General	Professional Career Duration	Lifetime Concussion History	1.00	0.52	0.50	0.52	0.48	0.00	0.52
General	Professional Career Duration	Age	0.00	0.05	0.23	0.05	0.95	0.00	0.95
General	Professional Career Duration	Highest Education	0.00	0.08	0.27	0.08	0.92	0.00	0.92
General	Professional Career Duration	Race	0.00	0.00	0.00	0.00	1.00	0.00	1.00
General	Lifetime Concussion History	Age	0.00	0.06	0.24	0.06	0.94	0.00	0.94
General	Lifetime Concussion History	Highest Education	0.00	0.07	0.26	0.07	0.93	0.00	0.93
General	Lifetime Concussion History	Race	0.00	0.00	0.00	0.00	1.00	0.00	1.00
General	Age	Highest Education	1.00	0.62	0.49	0.62	0.38	0.00	0.62
General	Age	Race	0.00	0.06	0.23	0.06	0.94	0.00	0.94
General	Highest Education	Race	0.00	0.02	0.14	0.02	0.98	0.00	0.98

Vocabulary									
**Dominance**	**i**	**j**	**Dij**	**mDij**	**SE.Dij**	**Pij**	**Pji**	**Pnoij**	**Rep**
Complete	Age of First Exposure	Lifetime Football Exposure	0.50	0.35	0.26	0.03	0.32	0.65	0.65
Complete	Age of First Exposure	Professional Career Duration	0.00	0.28	0.25	0.01	0.44	0.56	0.44
Complete	Age of First Exposure	Lifetime Concussion History	0.50	0.47	0.17	0.03	0.09	0.89	0.89
Complete	Age of First Exposure	Age	0.00	0.20	0.25	0.00	0.61	0.39	0.61
Complete	Age of First Exposure	Highest Education	0.00	0.04	0.14	0.00	0.92	0.08	0.92
Complete	Age of First Exposure	Race	0.00	0.00	0.00	0.00	1.00	0.00	1.00
Complete	Lifetime Football Exposure	Professional Career Duration	0.00	0.41	0.22	0.02	0.20	0.78	0.20
Complete	Lifetime Football Exposure	Lifetime Concussion History	0.50	0.54	0.18	0.10	0.03	0.87	0.87
Complete	Lifetime Football Exposure	Age	0.00	0.29	0.26	0.01	0.43	0.55	0.43
Complete	Lifetime Football Exposure	Highest Education	0.00	0.07	0.18	0.01	0.87	0.13	0.87
Complete	Lifetime Football Exposure	Race	0.00	0.00	0.00	0.00	1.00	0.00	1.00
Complete	Professional Career Duration	Lifetime Concussion History	0.50	0.66	0.25	0.34	0.01	0.65	0.65
Complete	Professional Career Duration	Age	0.50	0.39	0.26	0.05	0.28	0.67	0.67
Complete	Professional Career Duration	Highest Education	0.00	0.14	0.23	0.01	0.73	0.27	0.73
Complete	Professional Career Duration	Race	0.00	0.00	0.00	0.00	1.00	0.00	1.00
Complete	Lifetime Concussion History	Age	0.50	0.28	0.25	0.00	0.45	0.55	0.55
Complete	Lifetime Concussion History	Highest Education	0.00	0.04	0.14	0.00	0.92	0.08	0.92
Complete	Lifetime Concussion History	Race	0.00	0.00	0.00	0.00	1.00	0.00	1.00
Complete	Age	Highest Education	0.00	0.21	0.31	0.07	0.65	0.28	0.65
Complete	Age	Race	0.00	0.01	0.07	0.00	0.98	0.02	0.98
Complete	Highest Education	Race	0.00	0.10	0.25	0.04	0.84	0.12	0.84
Conditional	Age of First Exposure	Lifetime Football Exposure	0.00	0.30	0.33	0.09	0.49	0.42	0.49
Conditional	Age of First Exposure	Professional Career Duration	0.00	0.18	0.27	0.03	0.67	0.30	0.67
Conditional	Age of First Exposure	Lifetime Concussion History	0.50	0.44	0.31	0.15	0.26	0.59	0.59
Conditional	Age of First Exposure	Age	0.00	0.11	0.22	0.01	0.79	0.20	0.79
Conditional	Age of First Exposure	Highest Education	0.00	0.03	0.12	0.00	0.94	0.06	0.94
Conditional	Age of First Exposure	Race	0.00	0.00	0.00	0.00	1.00	0.00	1.00
Conditional	Lifetime Football Exposure	Professional Career Duration	0.00	0.27	0.32	0.08	0.55	0.37	0.55
Conditional	Lifetime Football Exposure	Lifetime Concussion History	0.50	0.61	0.33	0.35	0.13	0.51	0.51
Conditional	Lifetime Football Exposure	Age	0.00	0.21	0.27	0.03	0.61	0.37	0.61
Conditional	Lifetime Football Exposure	Highest Education	0.00	0.06	0.19	0.02	0.91	0.07	0.91
Conditional	Lifetime Football Exposure	Race	0.00	0.00	0.00	0.00	1.00	0.00	1.00
Conditional	Professional Career Duration	Lifetime Concussion History	1.00	0.86	0.28	0.77	0.06	0.17	0.77
Conditional	Professional Career Duration	Age	0.50	0.37	0.30	0.09	0.34	0.57	0.57
Conditional	Professional Career Duration	Highest Education	0.00	0.10	0.25	0.05	0.86	0.10	0.86
Conditional	Professional Career Duration	Race	0.00	0.00	0.00	0.00	1.00	0.00	1.00
Conditional	Lifetime Concussion History	Age	0.00	0.18	0.26	0.01	0.65	0.34	0.65
Conditional	Lifetime Concussion History	Highest Education	0.00	0.03	0.13	0.01	0.94	0.05	0.94

Conditional	Lifetime Concussion History	Race	0.00	0.00	0.00	0.00	1.00	0.00	1.00
Conditional	Age	Highest Education	0.00	0.21	0.35	0.12	0.70	0.18	0.70
Conditional	Age	Race	0.00	0.01	0.06	0.00	0.99	0.01	0.99
Conditional	Highest Education	Race	0.00	0.09	0.25	0.06	0.89	0.06	0.89
General	Age of First Exposure	Lifetime Football Exposure	0.00	0.21	0.40	0.21	0.79	0.00	0.79
General	Age of First Exposure	Professional Career Duration	0.00	0.10	0.30	0.10	0.90	0.00	0.90
General	Age of First Exposure	Lifetime Concussion History	0.00	0.33	0.47	0.33	0.67	0.00	0.67
General	Age of First Exposure	Age	0.00	0.04	0.19	0.04	0.96	0.00	0.96
General	Age of First Exposure	Highest Education	0.00	0.01	0.09	0.01	0.99	0.00	0.99
General	Age of First Exposure	Race	0.00	0.00	0.00	0.00	1.00	0.00	1.00
General	Lifetime Football Exposure	Professional Career Duration	0.00	0.25	0.43	0.25	0.75	0.00	0.75
General	Lifetime Football Exposure	Lifetime Concussion History	0.00	0.70	0.46	0.70	0.30	0.00	0.30
General	Lifetime Football Exposure	Age	0.00	0.09	0.29	0.09	0.91	0.00	0.91
General	Lifetime Football Exposure	Highest Education	0.00	0.04	0.20	0.04	0.96	0.00	0.96
General	Lifetime Football Exposure	Race	0.00	0.00	0.00	0.00	1.00	0.00	1.00
General	Professional Career Duration	Lifetime Concussion History	1.00	0.88	0.33	0.88	0.12	0.00	0.88
General	Professional Career Duration	Age	0.00	0.25	0.43	0.25	0.75	0.00	0.75
General	Professional Career Duration	Highest Education	0.00	0.07	0.25	0.07	0.94	0.00	0.94
General	Professional Career Duration	Race	0.00	0.00	0.00	0.00	1.00	0.00	1.00
General	Lifetime Concussion History	Age	0.00	0.05	0.23	0.05	0.95	0.00	0.95
General	Lifetime Concussion History	Highest Education	0.00	0.02	0.13	0.02	0.98	0.00	0.98
General	Lifetime Concussion History	Race	0.00	0.00	0.00	0.00	1.00	0.00	1.00
General	Age	Highest Education	0.00	0.21	0.41	0.21	0.79	0.00	0.79
General	Age	Race	0.00	0.00	0.04	0.00	1.00	0.00	1.00
General	Highest Education	Race	0.00	0.09	0.28	0.09	0.91	0.00	0.91
